# Correction: Scrutinized lipid utilization disrupts Amphotericin-B responsiveness in clinical isolates of *Leishmania donovani*

**DOI:** 10.7554/eLife.108677

**Published:** 2025-08-12

**Authors:** Supratim Pradhan, Dhruba Dhar, Debolina Manna, Shubhangi Chakraborty, Arkapriya Bhattacharyya, Khushi Chauhan, Rimi Mukherjee, Abhik Sen, Krishna Pandey, Soumen Das, Budhaditya Mukherjee

**Keywords:** Mouse

Pradhan S, Dhar D, Manna D, Chakraborty S, Bhattacharyya A, Chauhan K, Mukherjee R, Sen A, Pandey K, Das S, Mukherjee B. 2025. Scrutinized lipid utilization disrupts Amphotericin-B responsiveness in clinical isolates of Leishmania donovani. *eLife* **14**:RP102857. doi: 10.7554/eLife.102857.

Published 27 May 2025

We would like to report an unintentional error in Figure 1—figure supplement 1, affecting panel C i (lower left LD-S^1^/24 hr p.i) and panel C ii (lower right LD-R^2^/24 hr p.i). These panels were intended to show representative images of infected macrophages derived from BALB/c mice. However, during assembly of this figure supplement we erroneously duplicated the images from Figure 2Bi that showed the LD-S and LD-R images of infected macrophages from C57BL/6 (BL6) mice.

This error occurred during the compilation of composite figure panels, which involved extensive rearrangement and reformatting across multiple submission rounds and revisions. Amidst these repeated adjustments, a copy-and-paste mistake led to incorrect insertion of BL6 macrophage images in place of BALB/c images. The similarity in morphology between the two cell types likely contributed to the error going unnoticed at the time of submission.

The discrepancy was later identified by the first author during internal figure indexing for his thesis. Upon re-examination of the original data, the error was confirmed, and we have now corrected it by replacing the incorrect BL6 macrophage images with the appropriate BALB/c macrophage images.

The revised Figure 1—figure supplement 1 now accurately displays the correct experimental conditions in panel C i, lower left, and panel C ii, lower right. We sincerely apologize for this oversight and any confusion it may have caused.

The legend for Figure 1—figure supplement 1 Ci and Cii it shown for reference: “(**C**) Representative Giemsa-stained images LD-infected-PEC, 4 and 24 hr p.i. for (i) LD-S and (ii) LD-R strains. Giemsa images are represented in gray scale to clearly represent LD nucleus (black arrow). Scale bar: 20 μM.”

The corrected Figure 1—figure supplement 1 with updated panel Ci and Cii is shown here:

**Figure 1:**
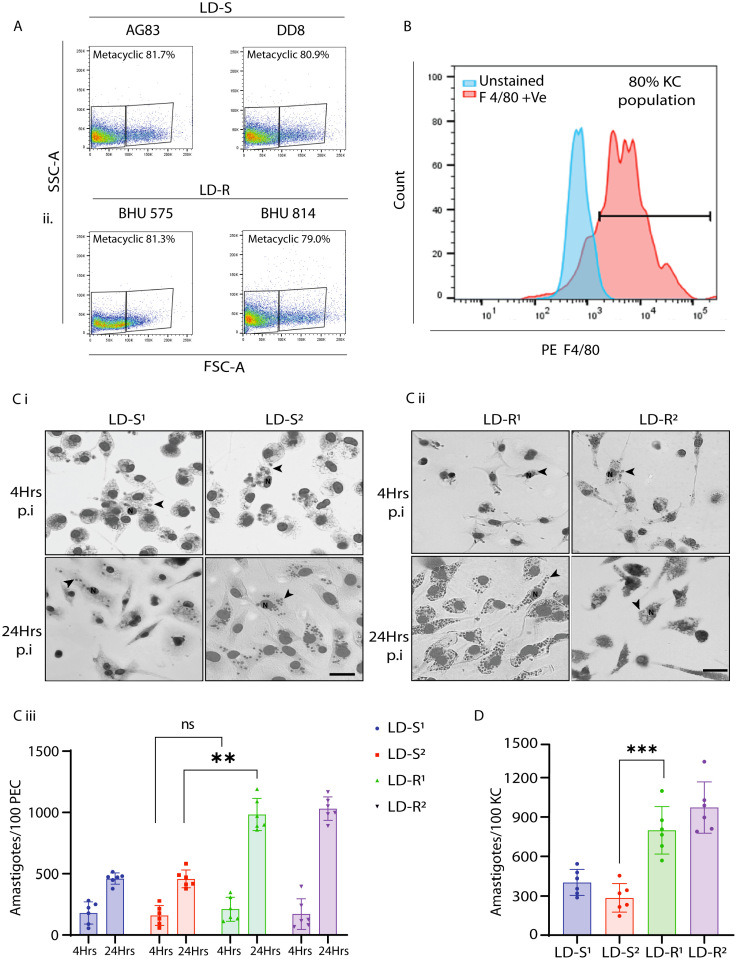


The originally published Figure 1—figure supplement 1 is shown for reference:

**Figure 2:**
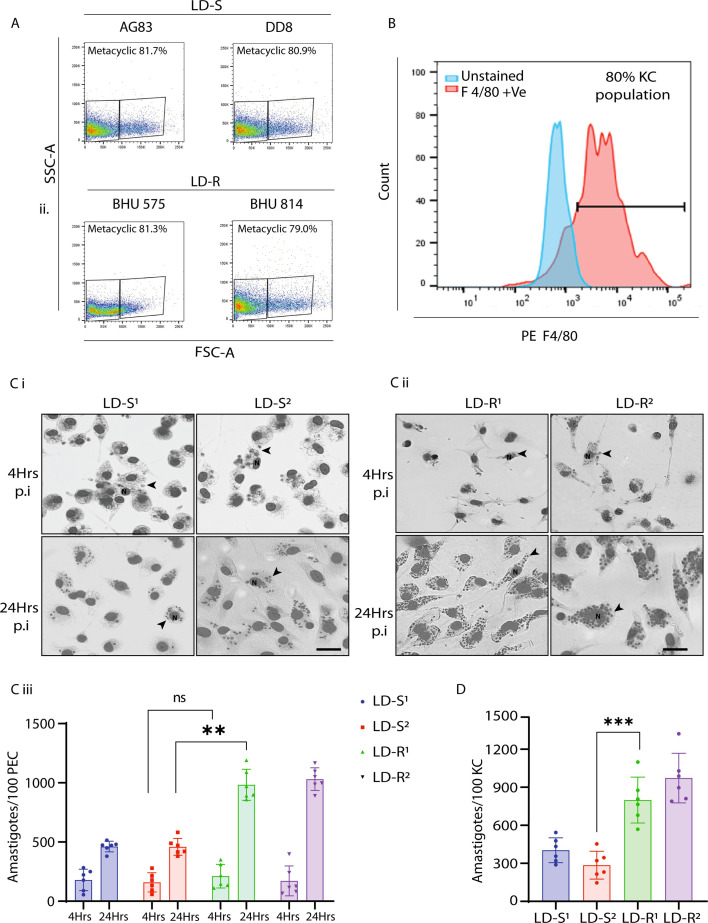


The article has been corrected accordingly.

