# Supplementary figures and images for: Scrutinized lipid utilization disrupts Amphotericin-B responsiveness in clinical isolates of Leishmania donovani
Source: eLife. 2025 May 27;14:RP102857. doi: 10.7554/eLife.102857 (PMC12113272; doi:10.7554/eLife.102857)

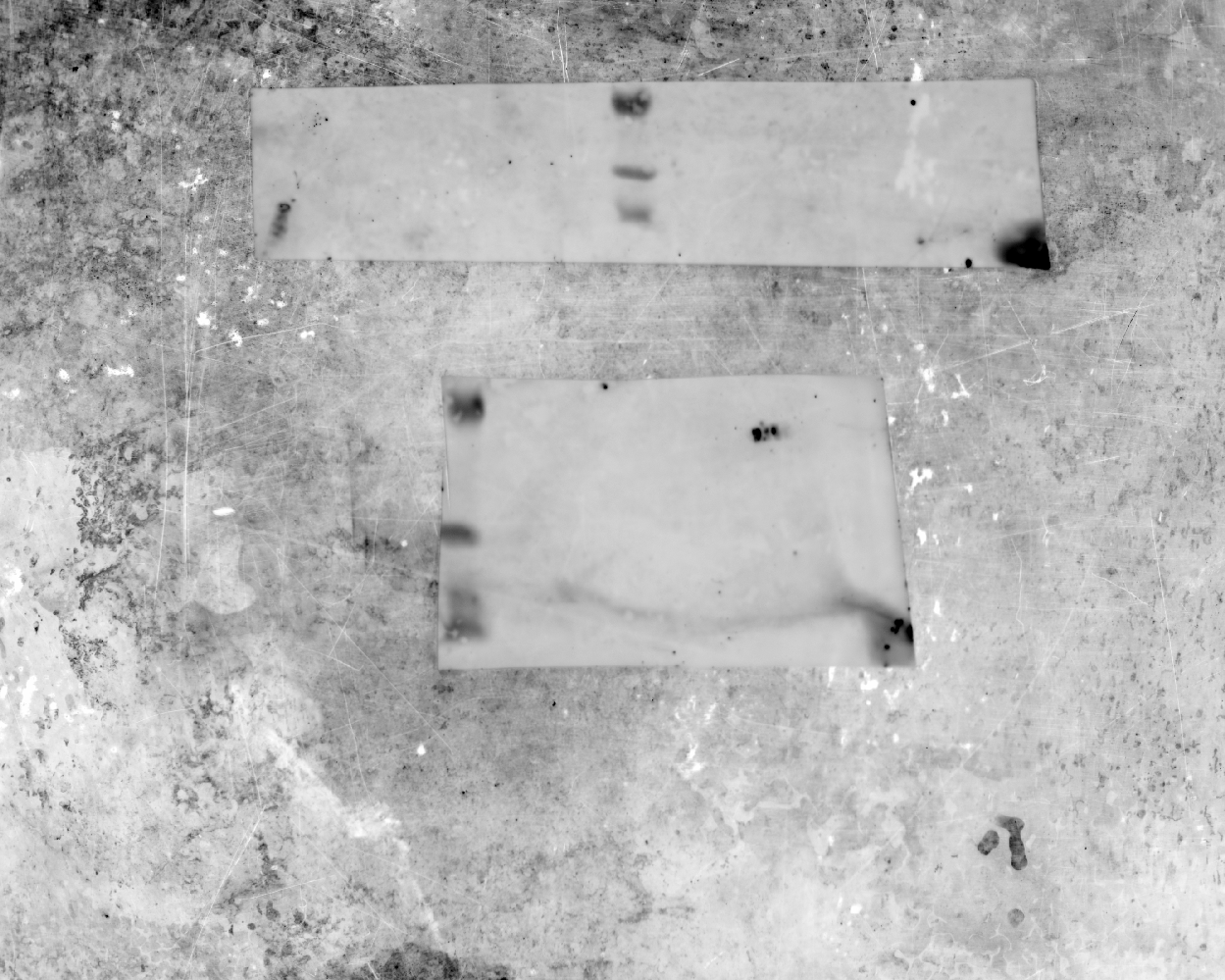

Supplement: Figure 3—source data 1. [file elife-102857-fig3-data1.zip › Figure 3 source data/Figure 3 Source data 1/KMP11 Marker.tif]

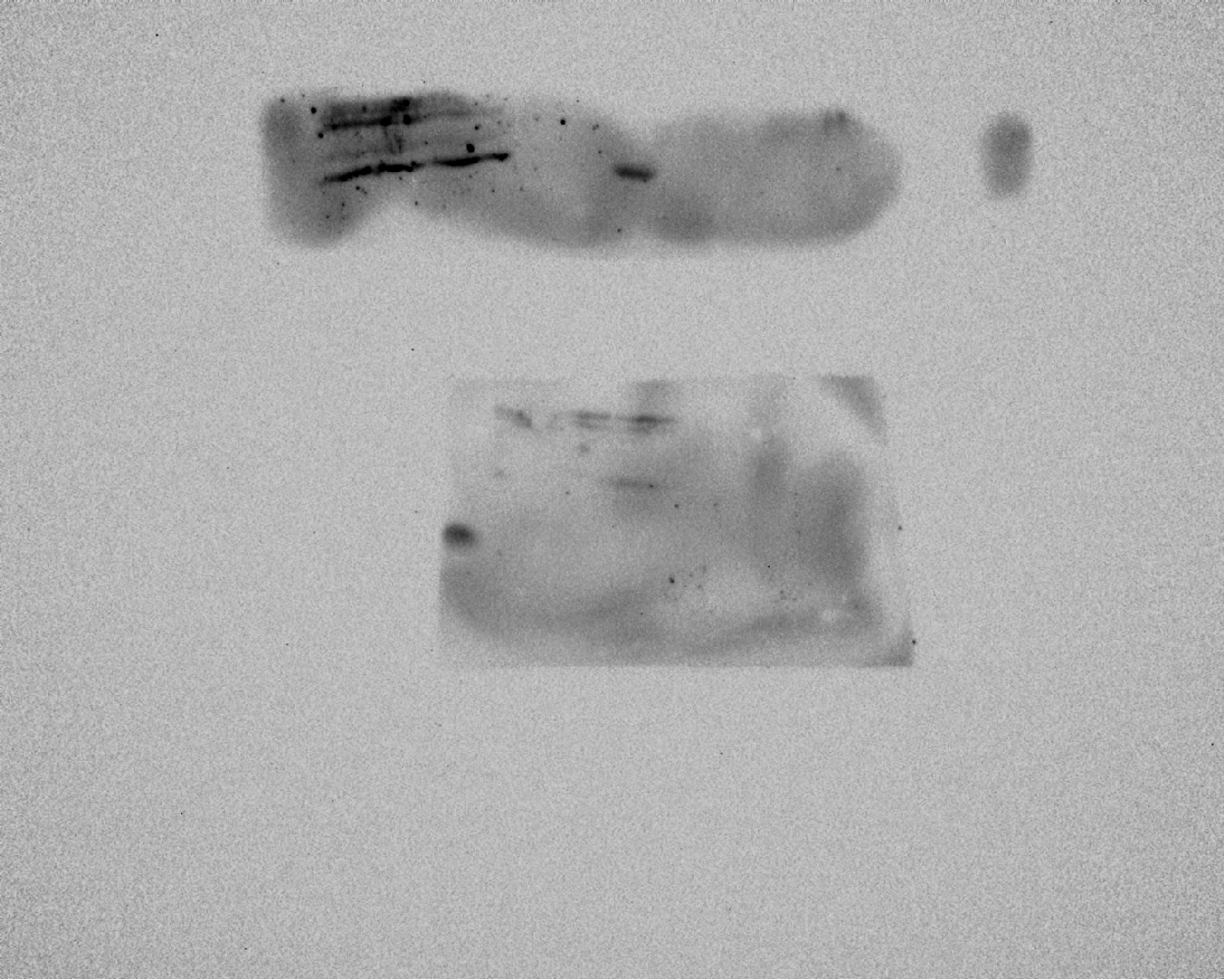

Supplement: Figure 3—source data 1. [file elife-102857-fig3-data1.zip › Figure 3 source data/Figure 3 Source data 1/kmp11.tif]

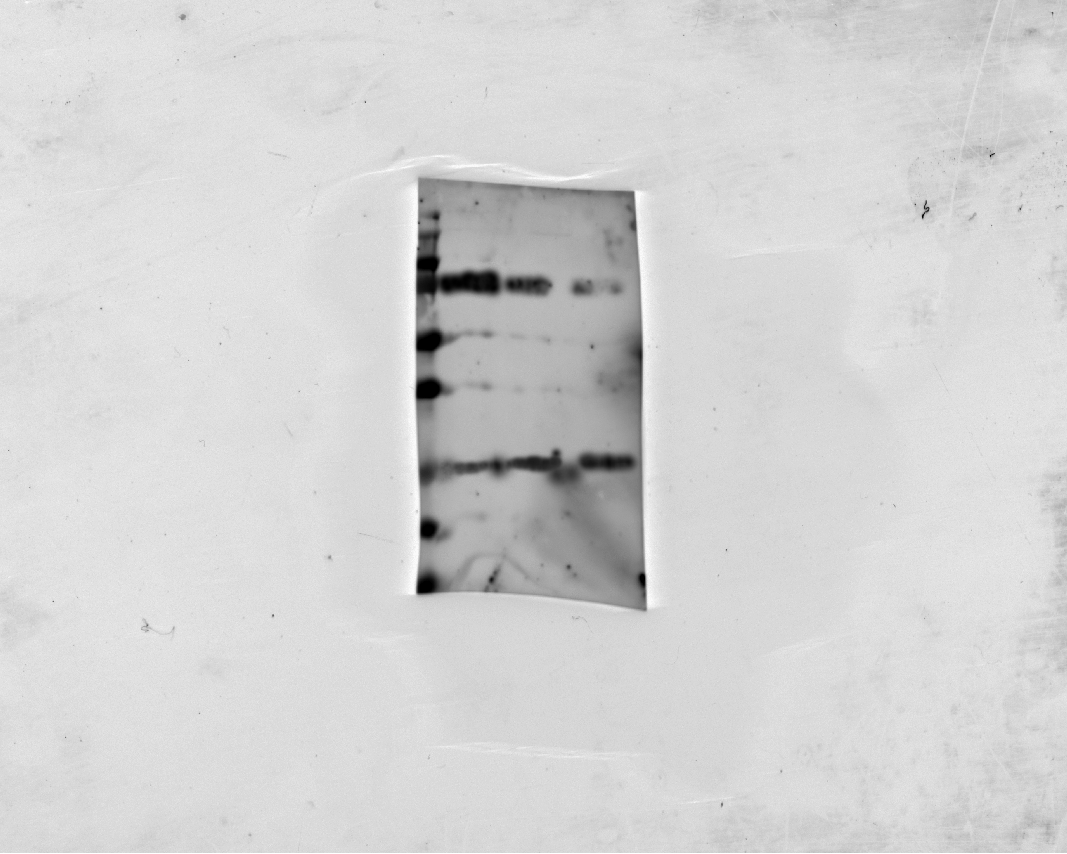

Supplement: Figure 3—source data 1. [file elife-102857-fig3-data1.zip › Figure 3 source data/Figure 3 Source data 1/pv lamp1_b-actin.tif]

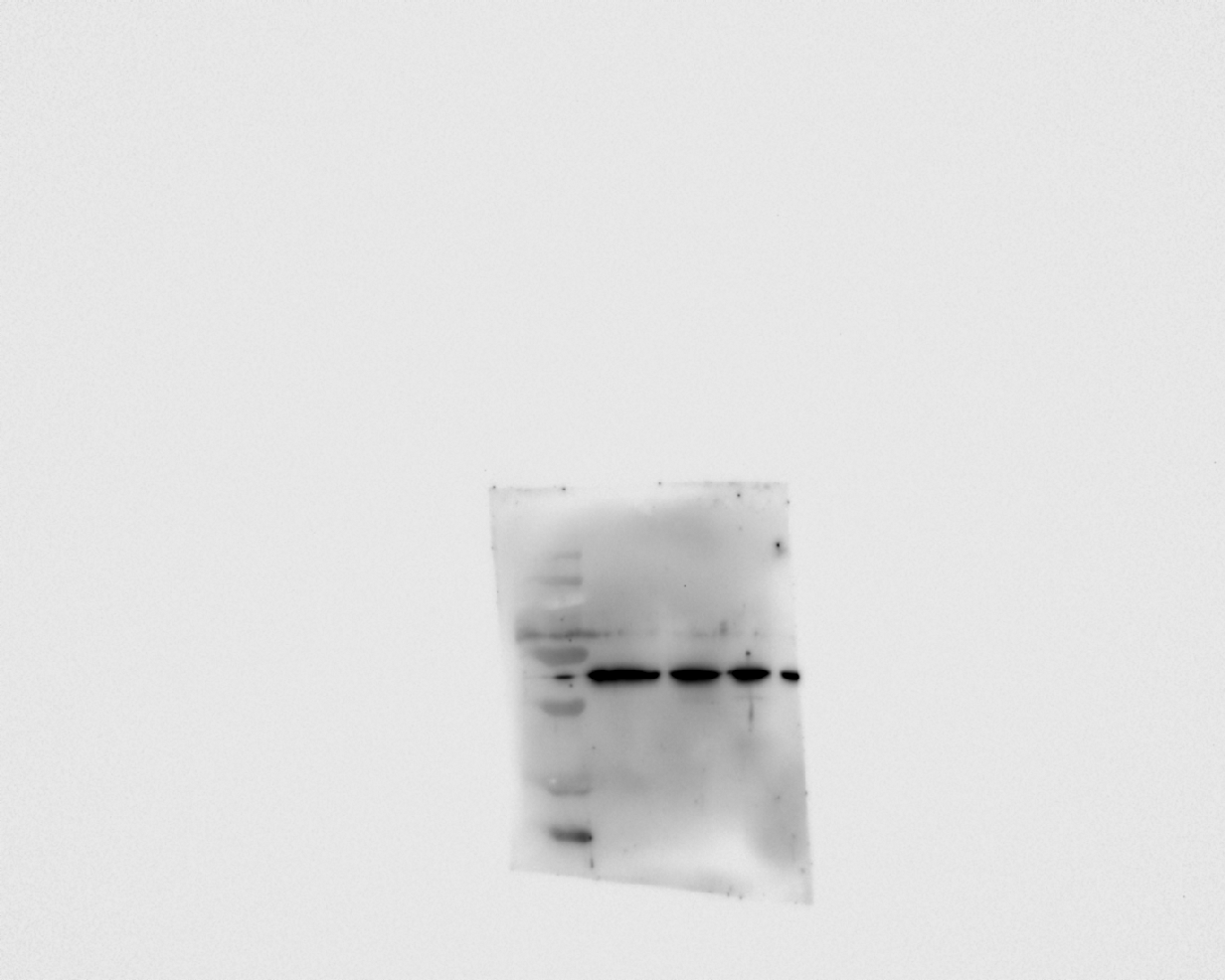

Supplement: Figure 3—source data 1. [file elife-102857-fig3-data1.zip › Figure 3 source data/Figure 3 source data 2/b actin whole cell lysate.tif]

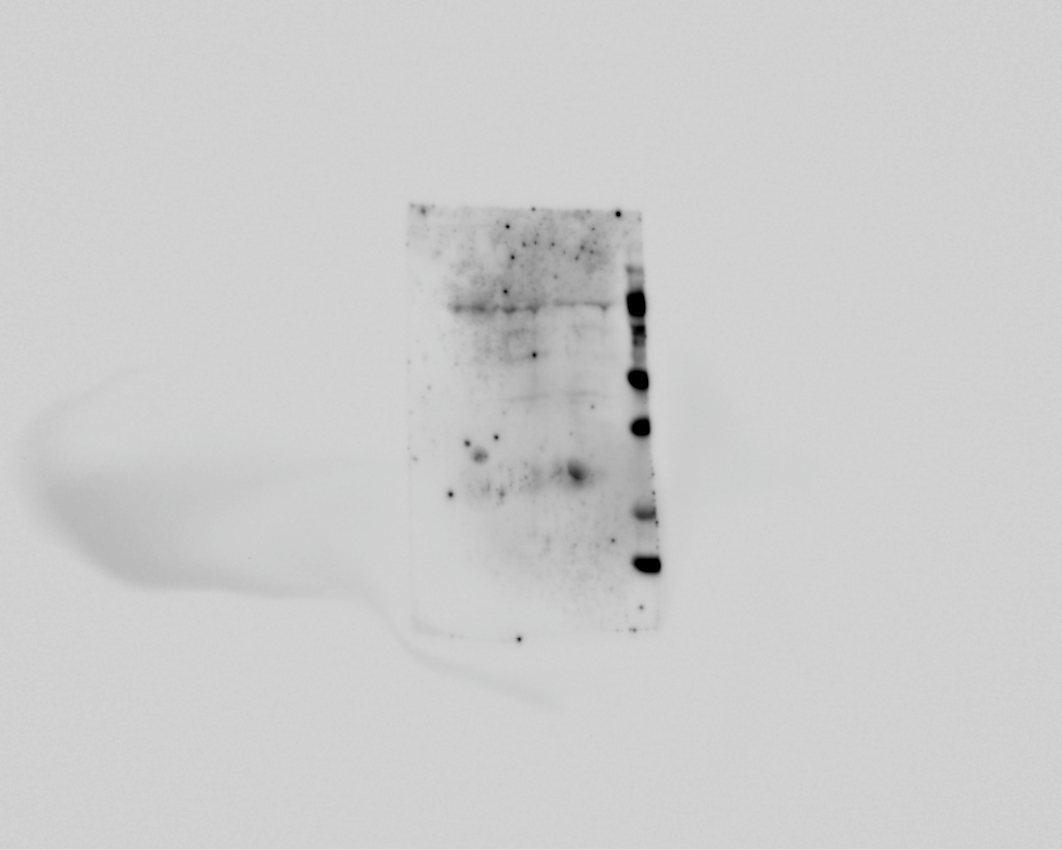

Supplement: Figure 3—source data 1. [file elife-102857-fig3-data1.zip › Figure 3 source data/Figure 3 source data 2/LAMP 1 whole cell lysate.tif]

Figure 3

C ii

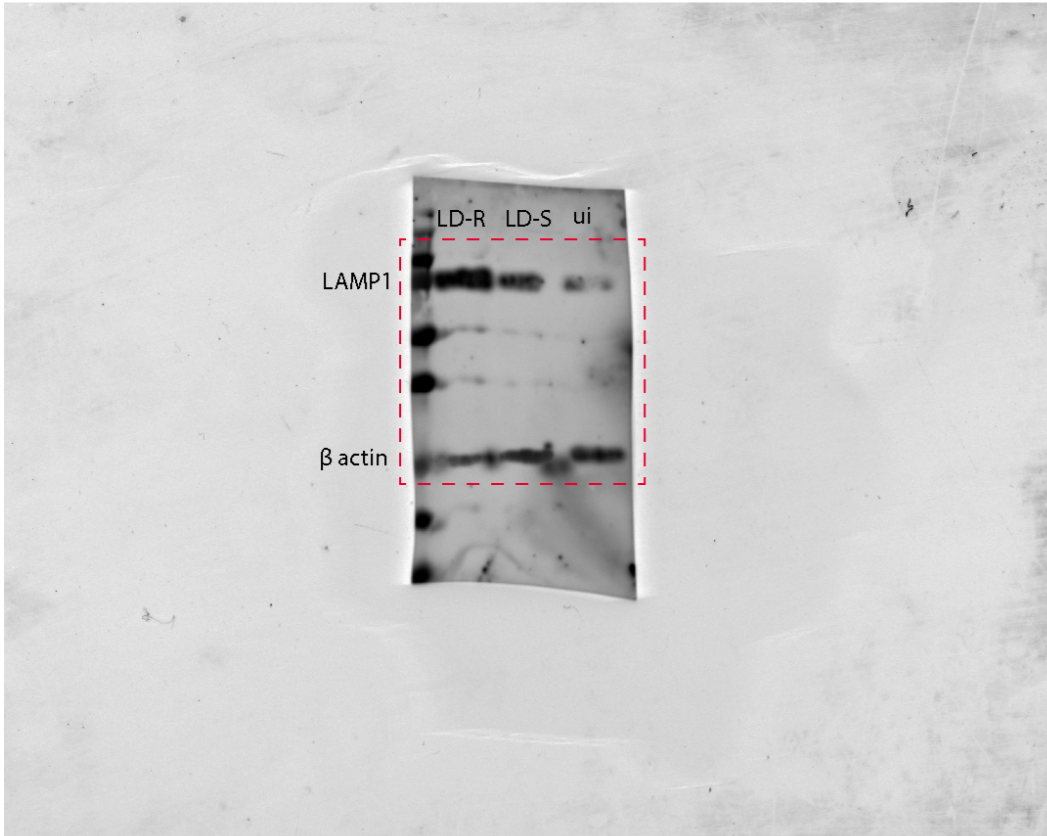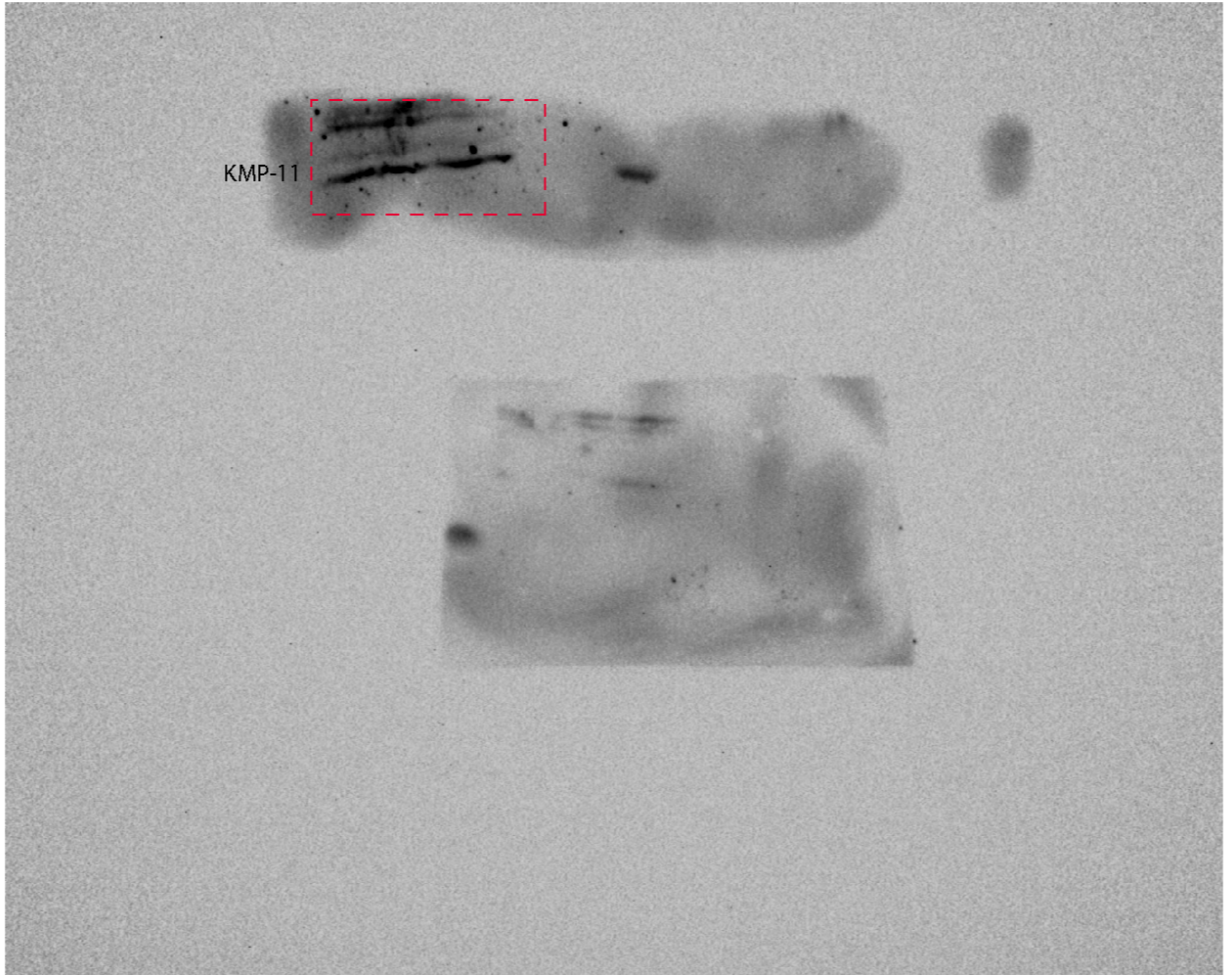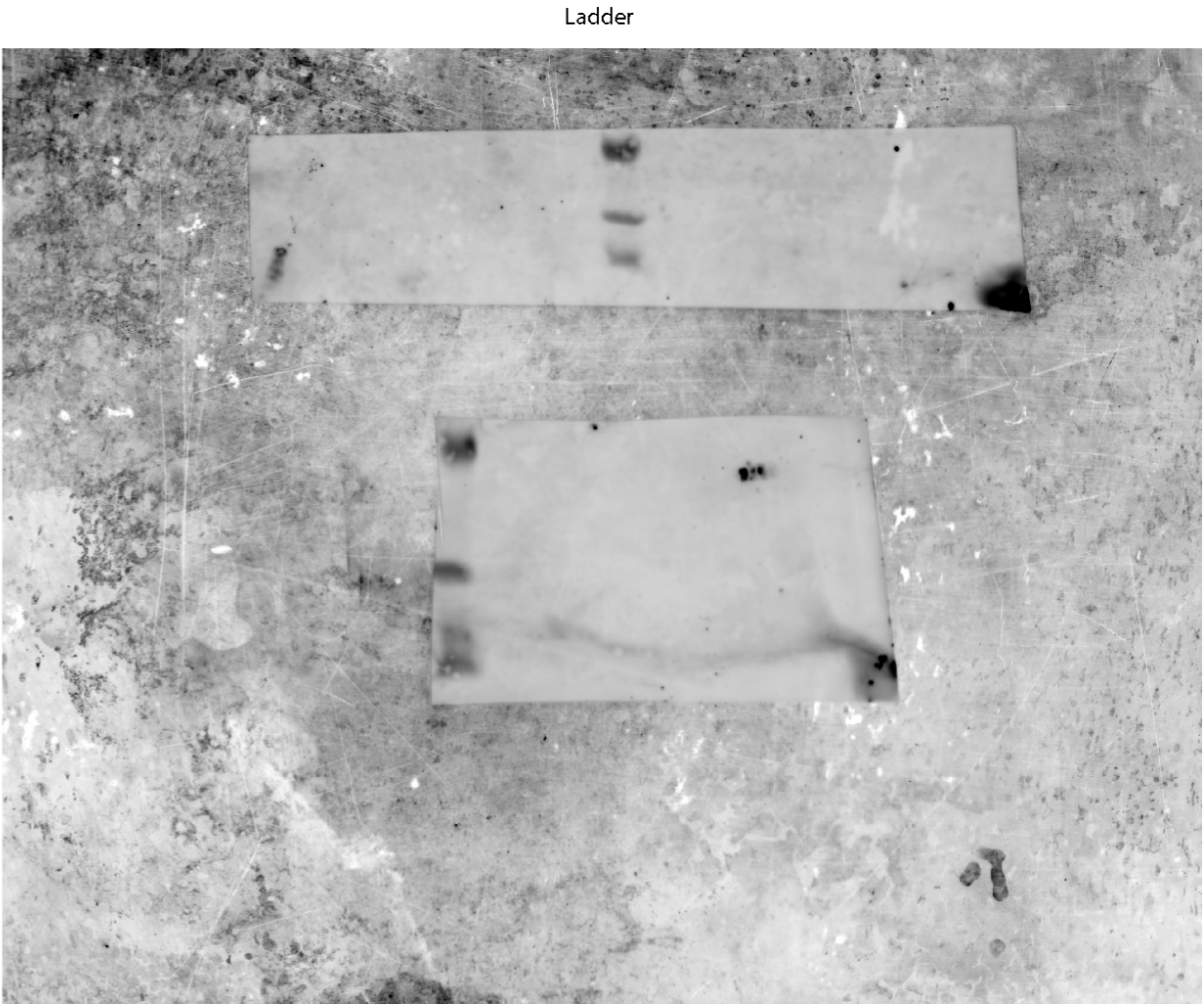

Supplement: Figure 3—source data 2. [file elife-102857-fig3-data2.zip › Figure 3 source data marked/Figure 3 source data 1 marked.pdf]

C iii

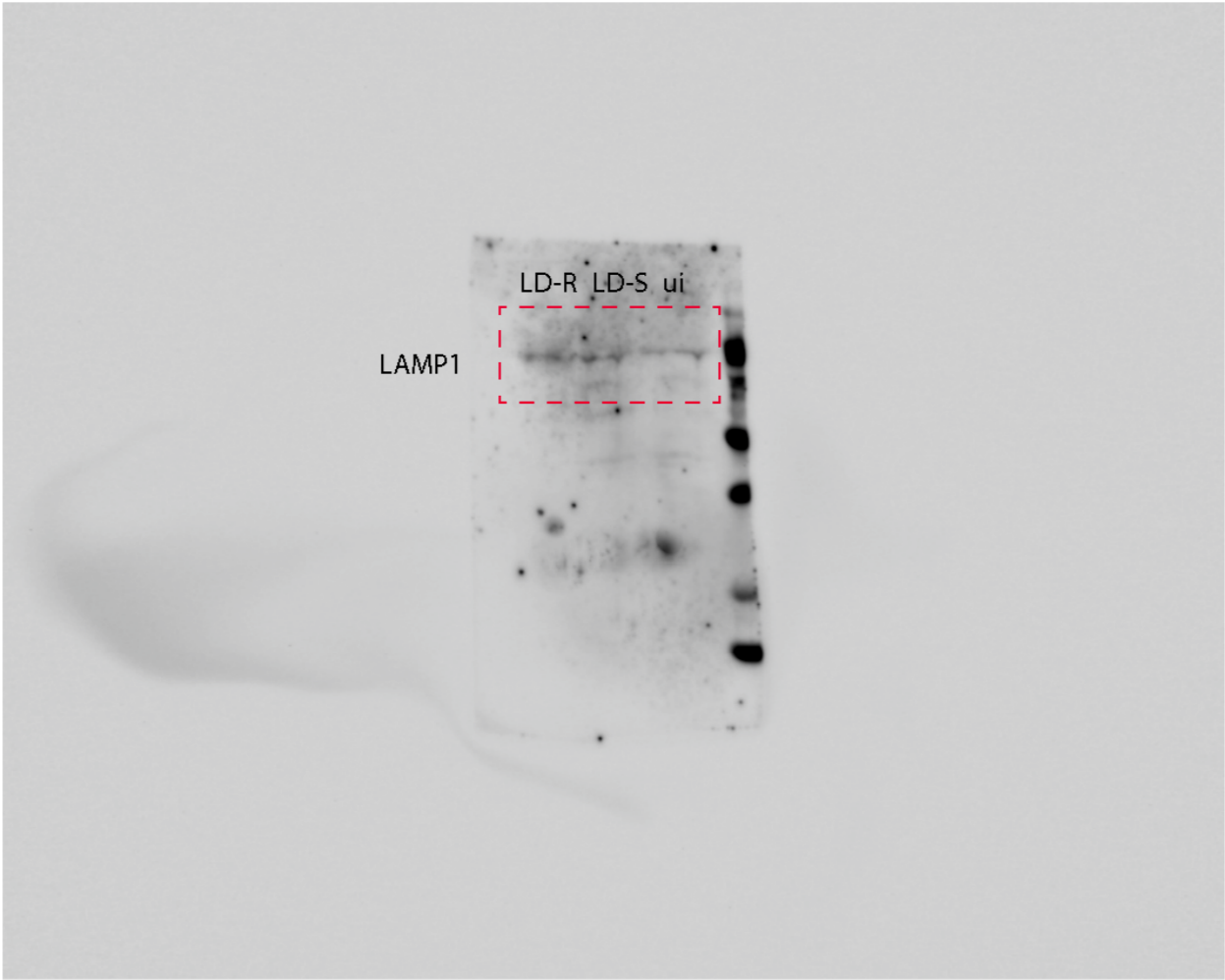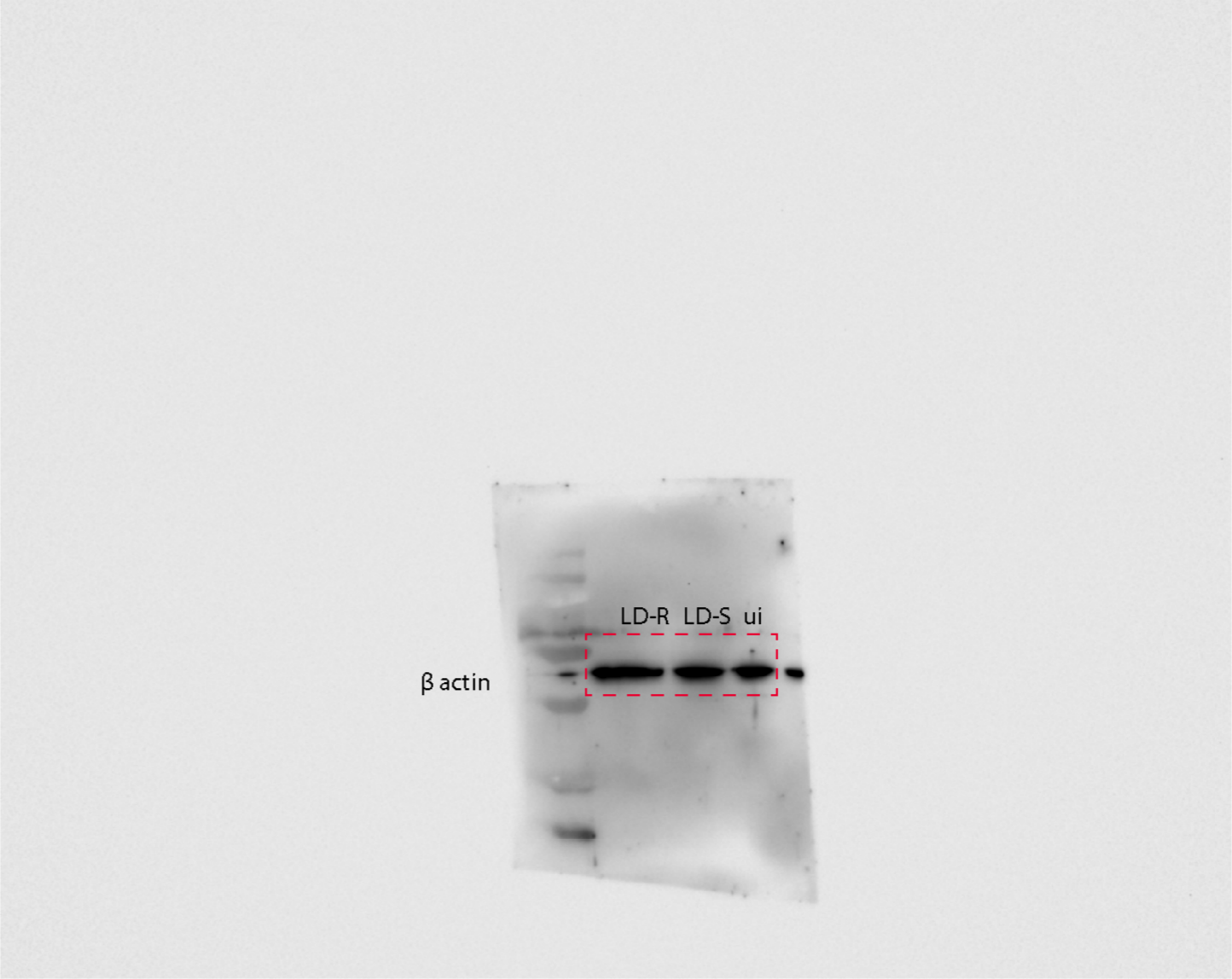

Supplement: Figure 3—source data 2. [file elife-102857-fig3-data2.zip › Figure 3 source data marked/Figure 3 source data 2 marked.pdf]

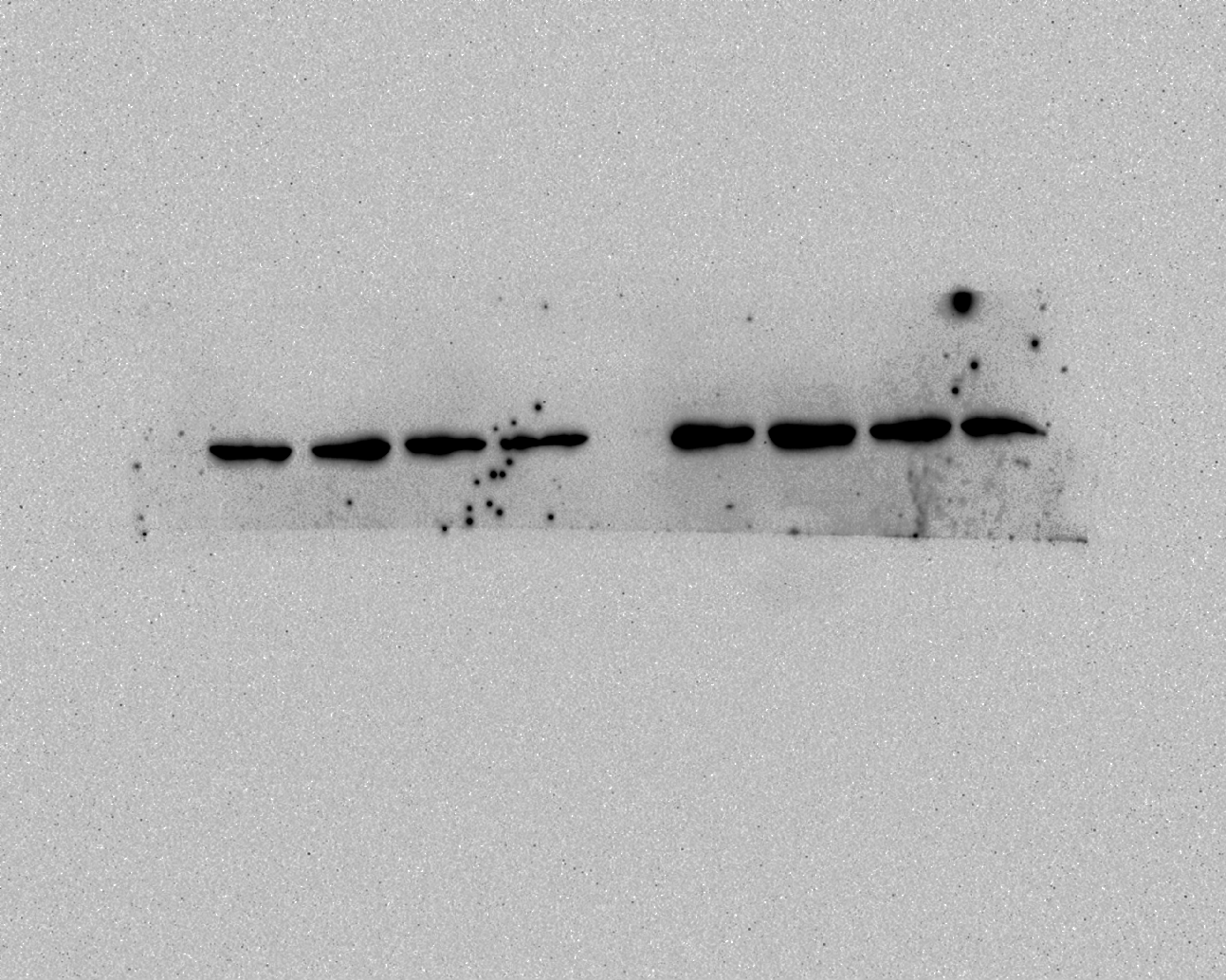

Supplement: Figure 4—source data 1. [file elife-102857-fig4-data1.zip › Figure 4 source data 1/b actin.tif]

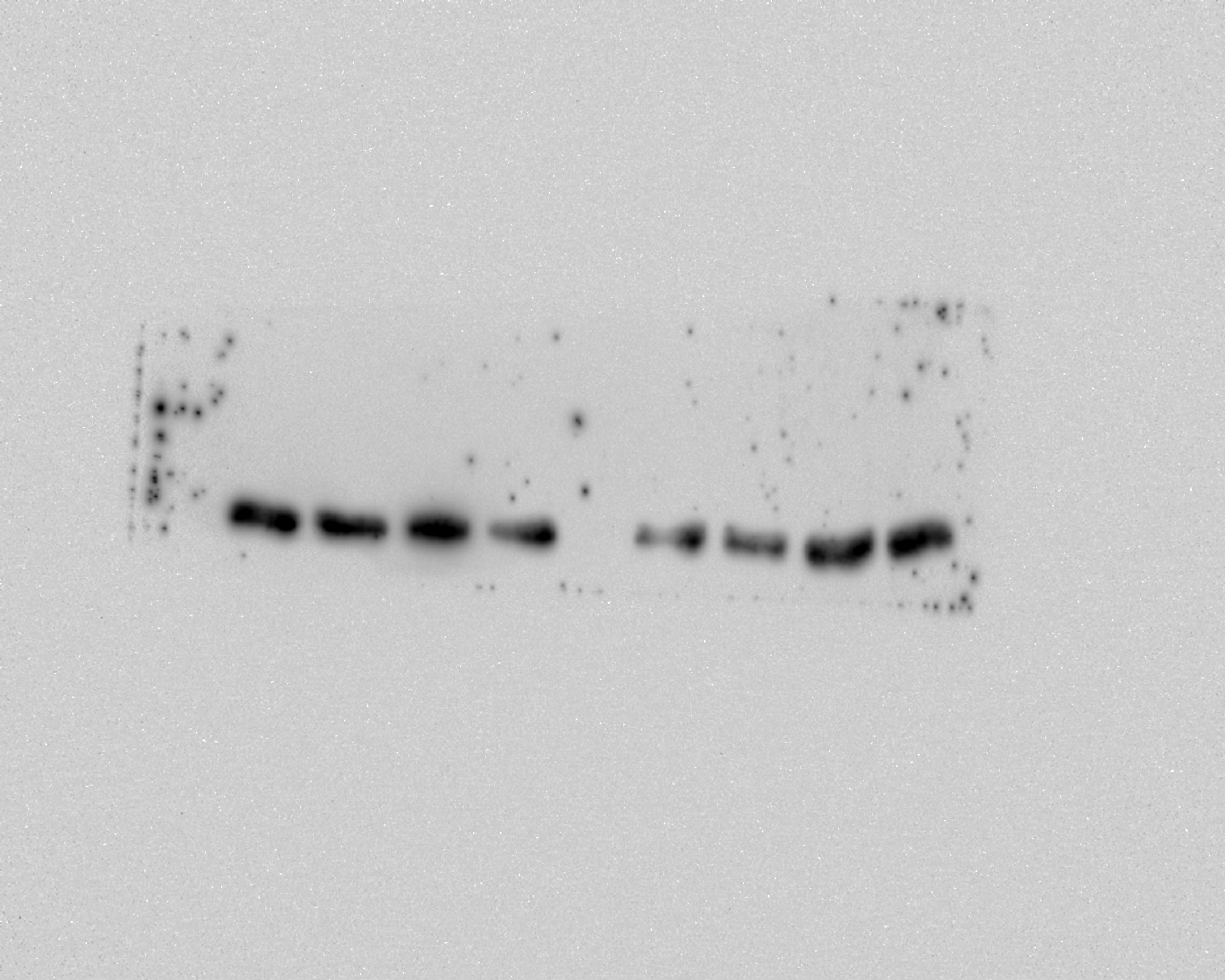

Supplement: Figure 4—source data 1. [file elife-102857-fig4-data1.zip › Figure 4 source data 1/coffilin.tif]

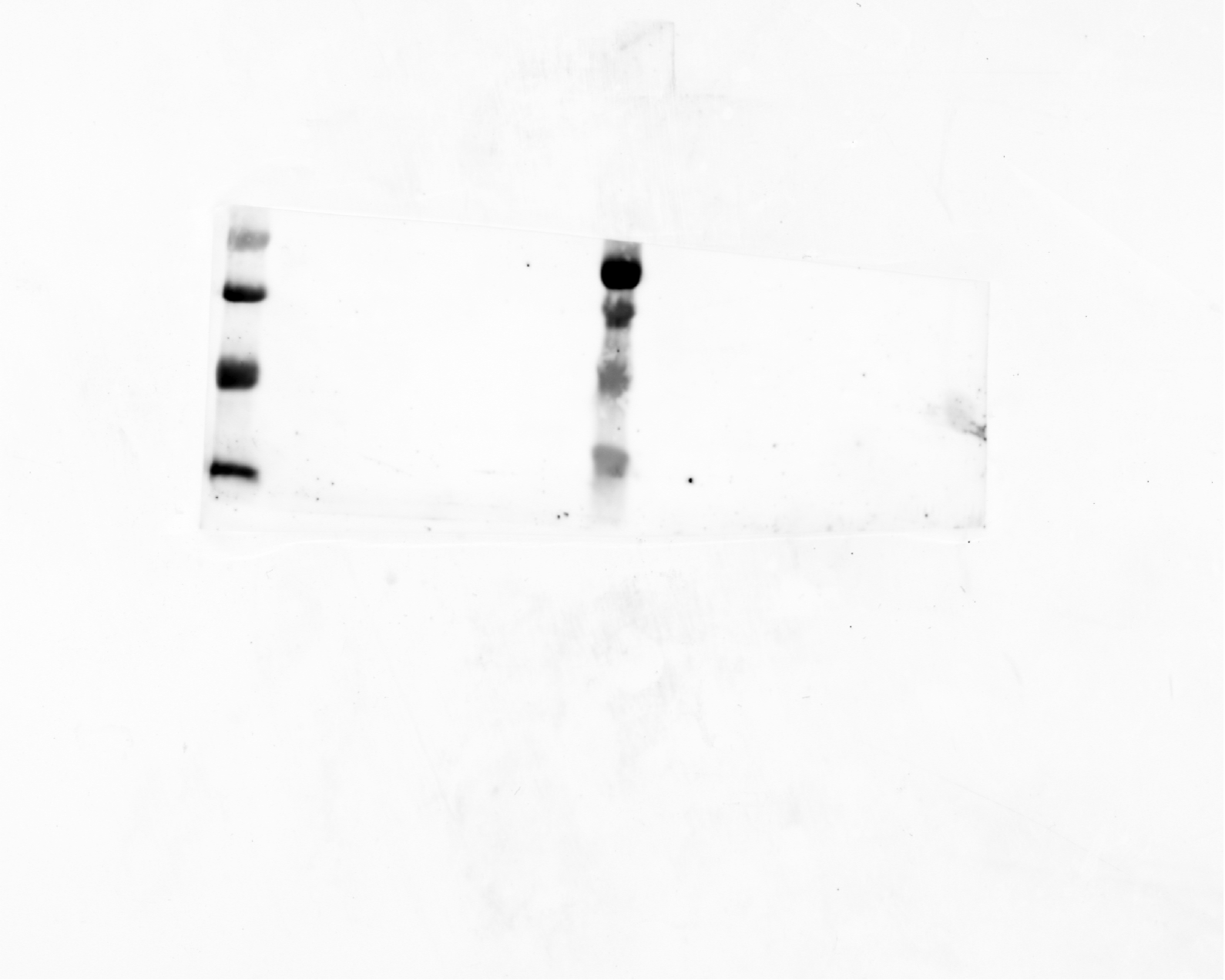

Supplement: Figure 4—source data 1. [file elife-102857-fig4-data1.zip › Figure 4 source data 1/Ladder.tif]

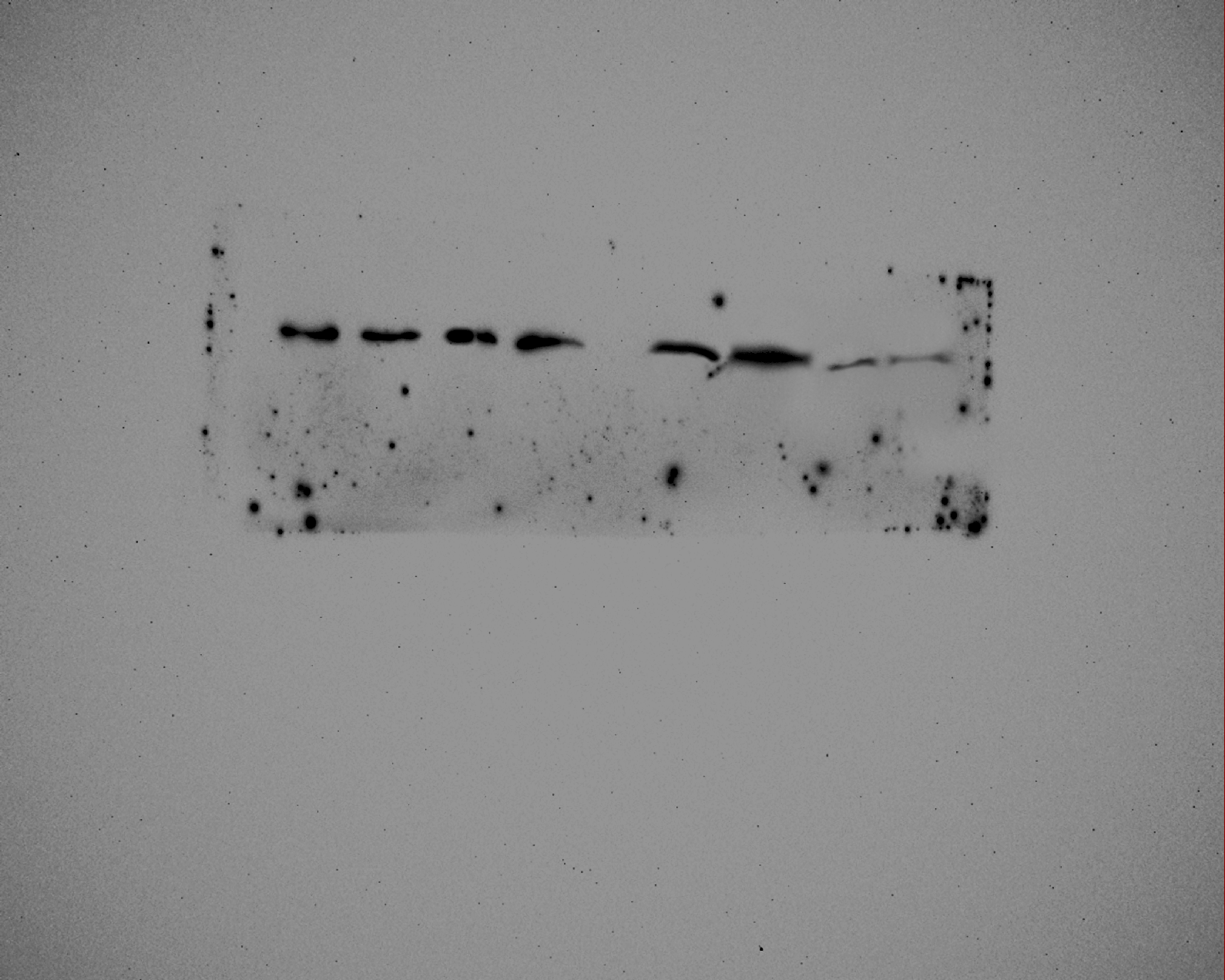

Supplement: Figure 4—source data 1. [file elife-102857-fig4-data1.zip › Figure 4 source data 1/p coffilin.tif]

Figure 4

B i

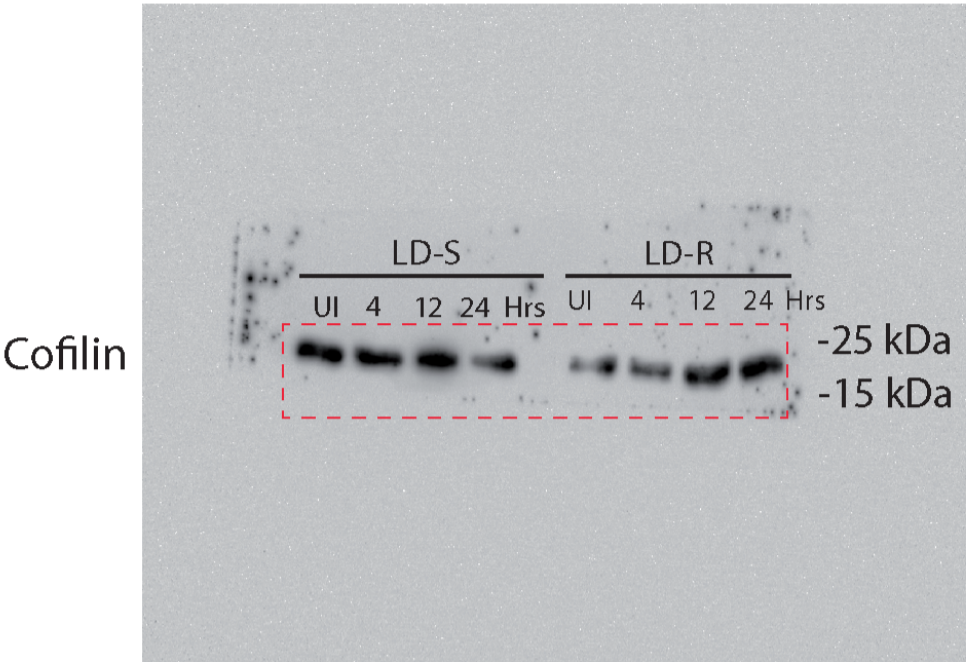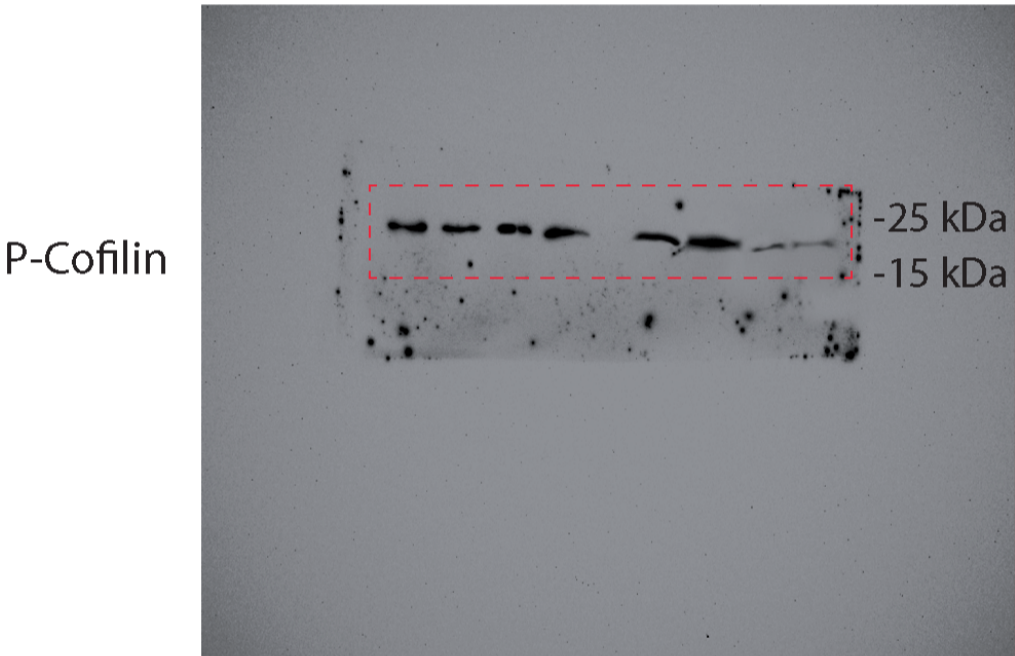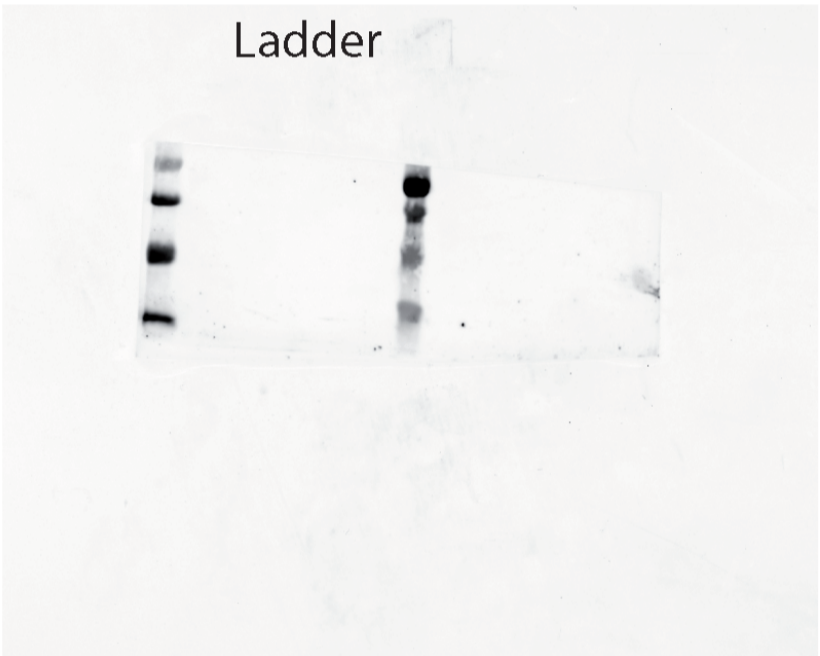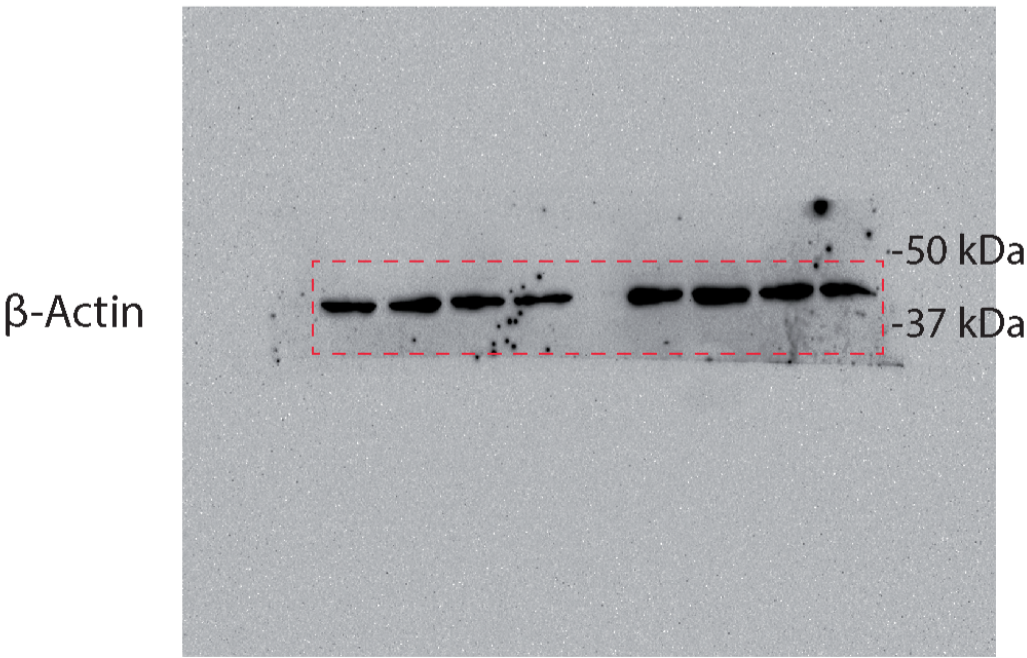

Supplement: Figure 4—source data 2. [file elife-102857-fig4-data2.zip › Figure 4 source data 1 marked/Figure 4 source data 1 marked.pdf]

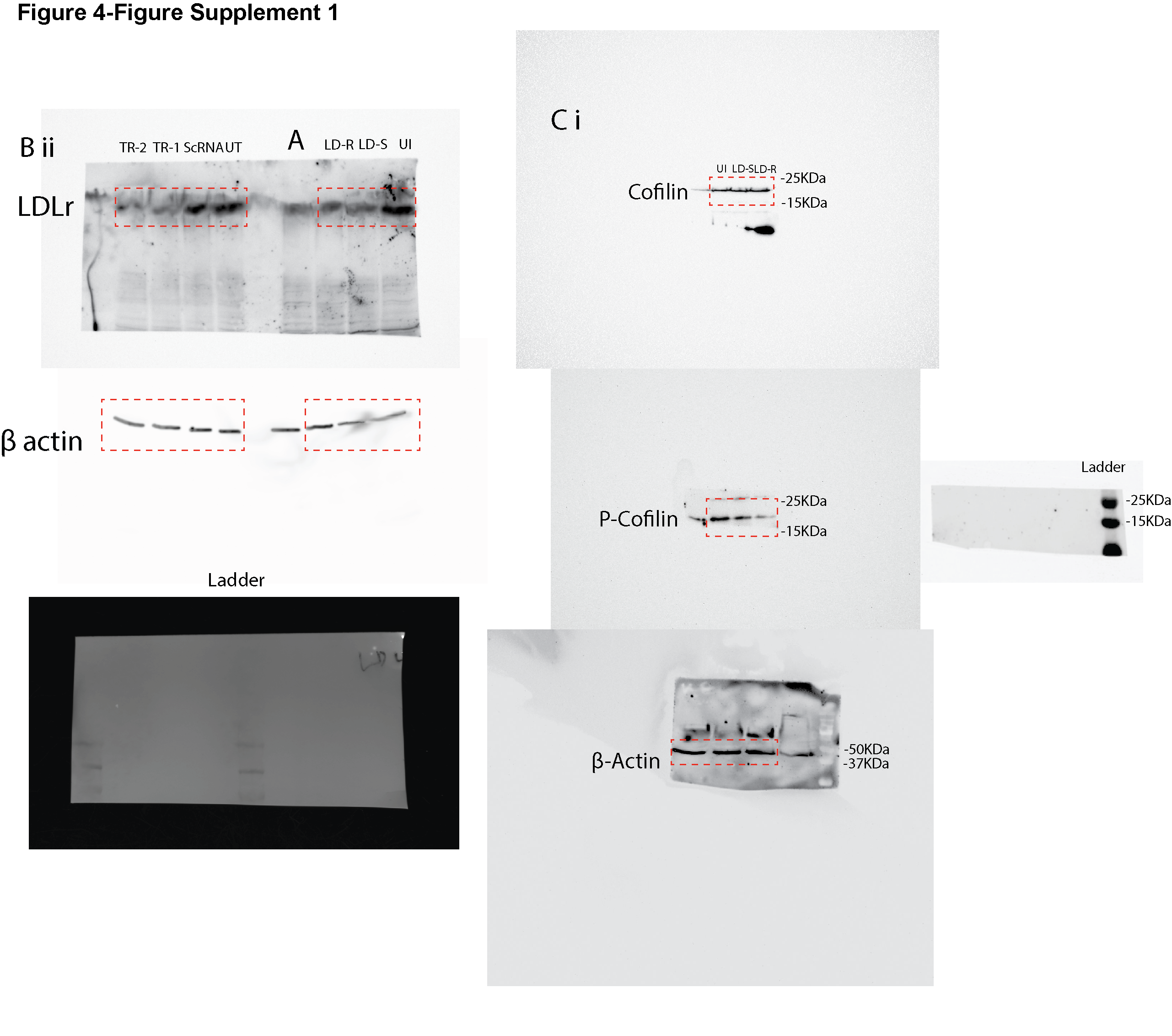

Supplement: Figure 4—figure supplement 1—source data 1. [file elife-102857-fig4-figsupp1-data1.zip › figure 4 supplementary 1 source data/Marked Figure 4 Figure supplement 1/Fig 4 Figure supplement 1.tif]

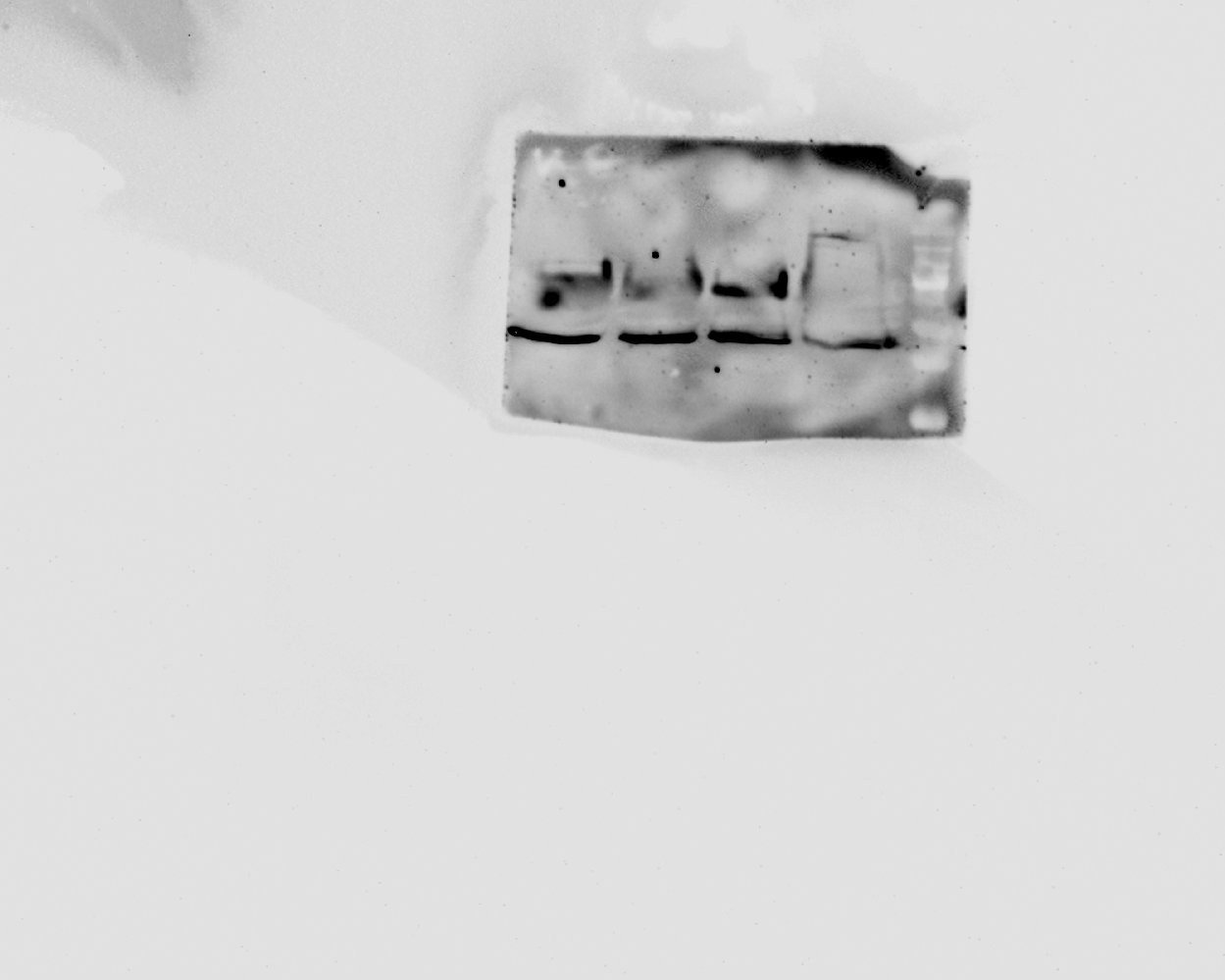

Supplement: Figure 4—figure supplement 1—source data 1. [file elife-102857-fig4-figsupp1-data1.zip › figure 4 supplementary 1 source data/Original file/b actin.tif]

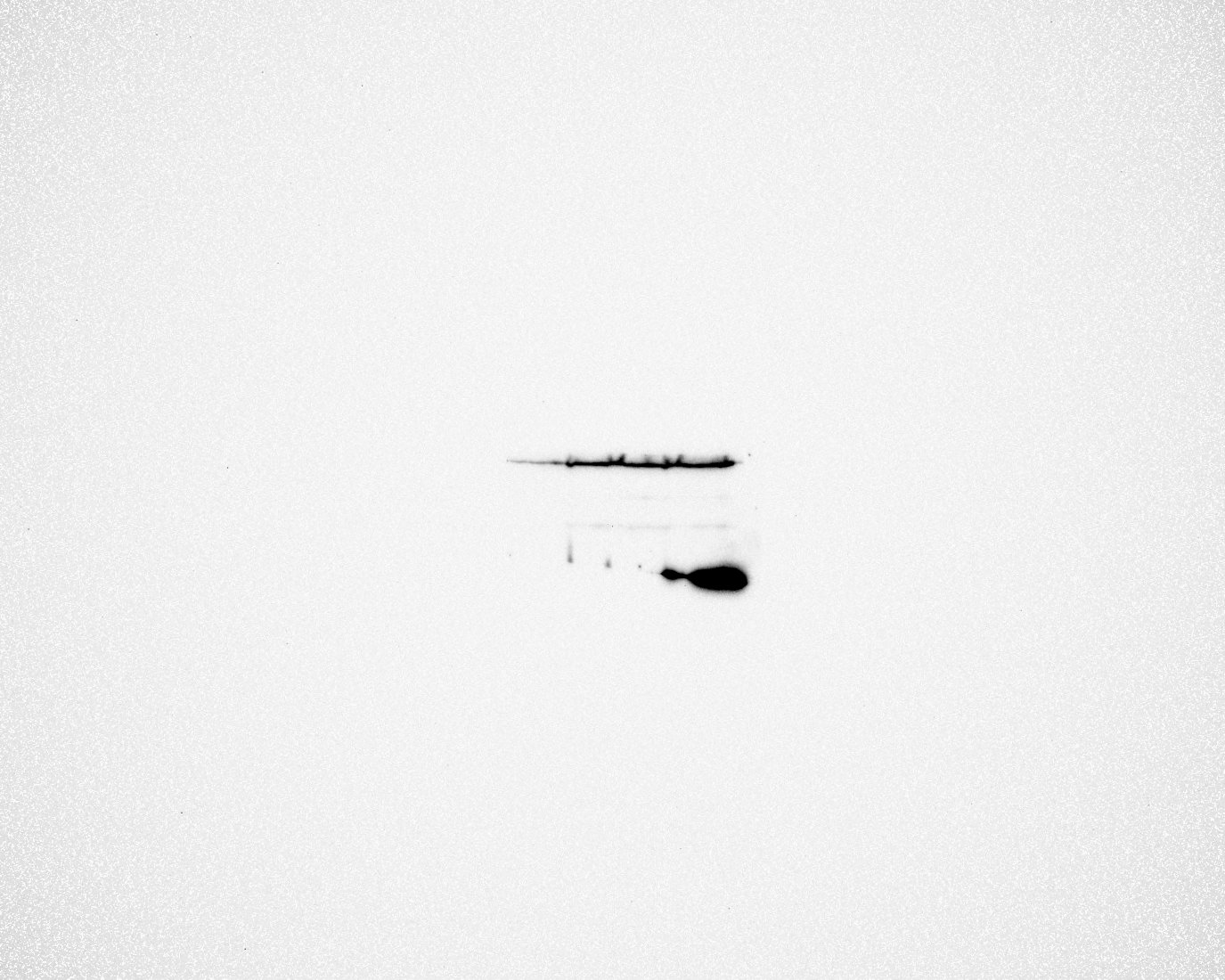

Supplement: Figure 4—figure supplement 1—source data 1. [file elife-102857-fig4-figsupp1-data1.zip › figure 4 supplementary 1 source data/Original file/coffilin.tif]

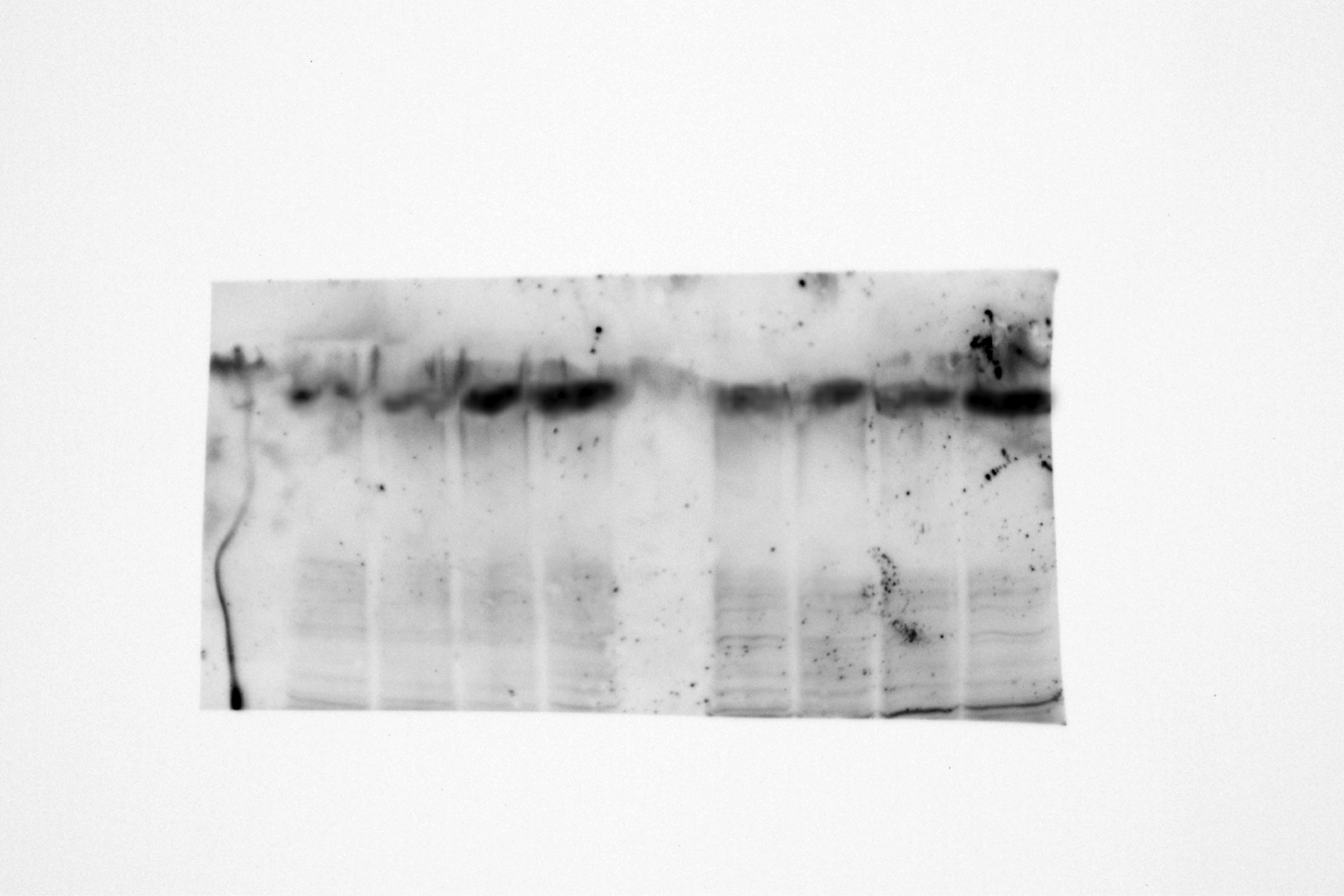

Supplement: Figure 4—figure supplement 1—source data 1. [file elife-102857-fig4-figsupp1-data1.zip › figure 4 supplementary 1 source data/Original file/Final LDL SiRNA and infection.tif]

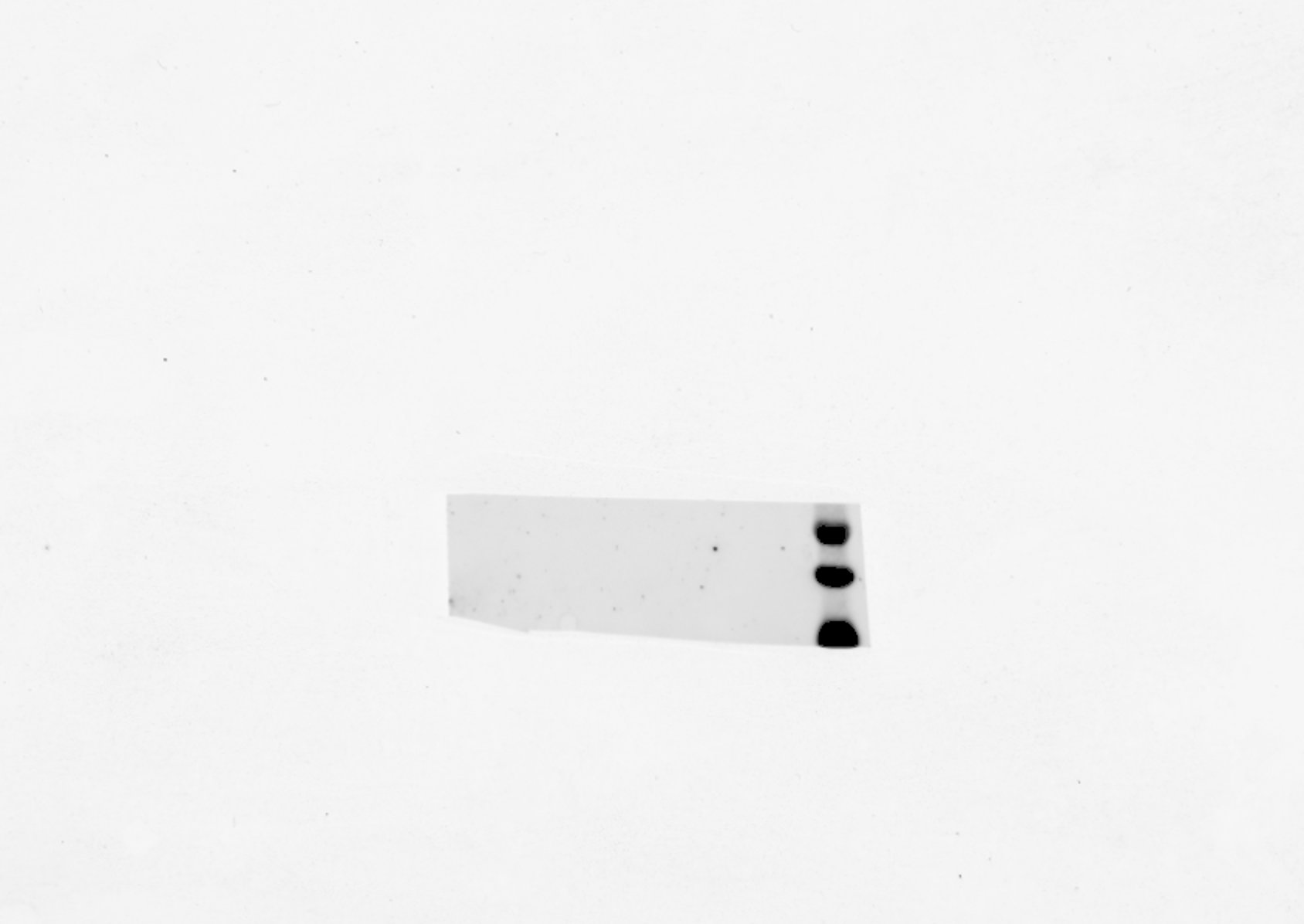

Supplement: Figure 4—figure supplement 1—source data 1. [file elife-102857-fig4-figsupp1-data1.zip › figure 4 supplementary 1 source data/Original file/p coffilin ladder.jpg]

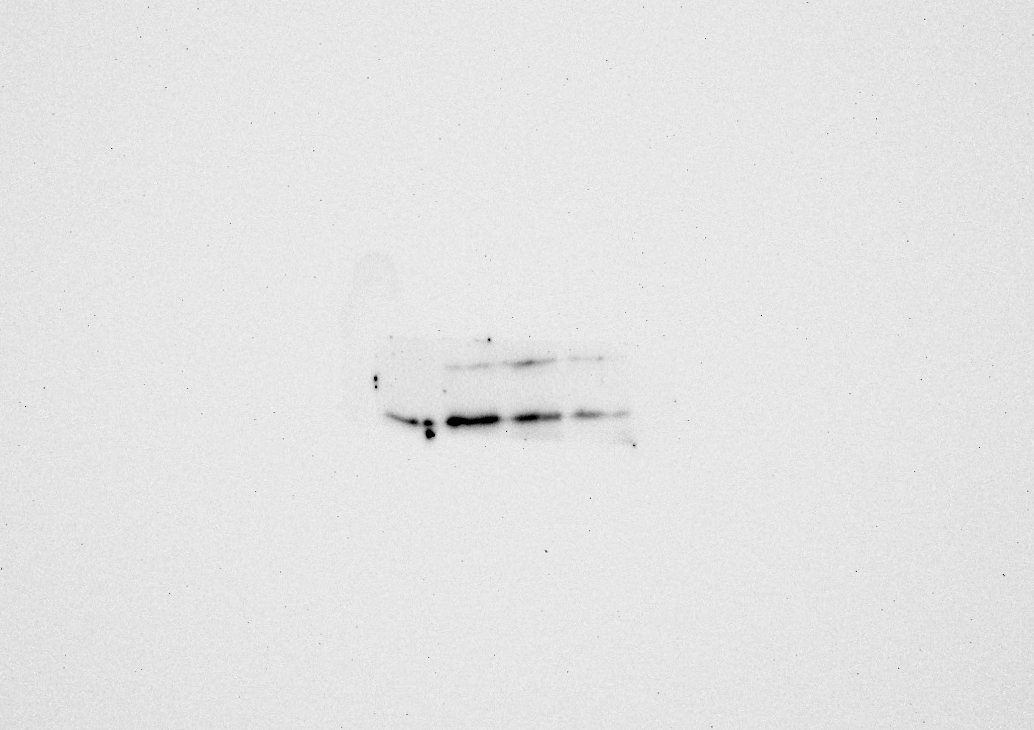

Supplement: Figure 4—figure supplement 1—source data 1. [file elife-102857-fig4-figsupp1-data1.zip › figure 4 supplementary 1 source data/Original file/p coffilin.jpg]

Figure 4-Figure Supplement 1

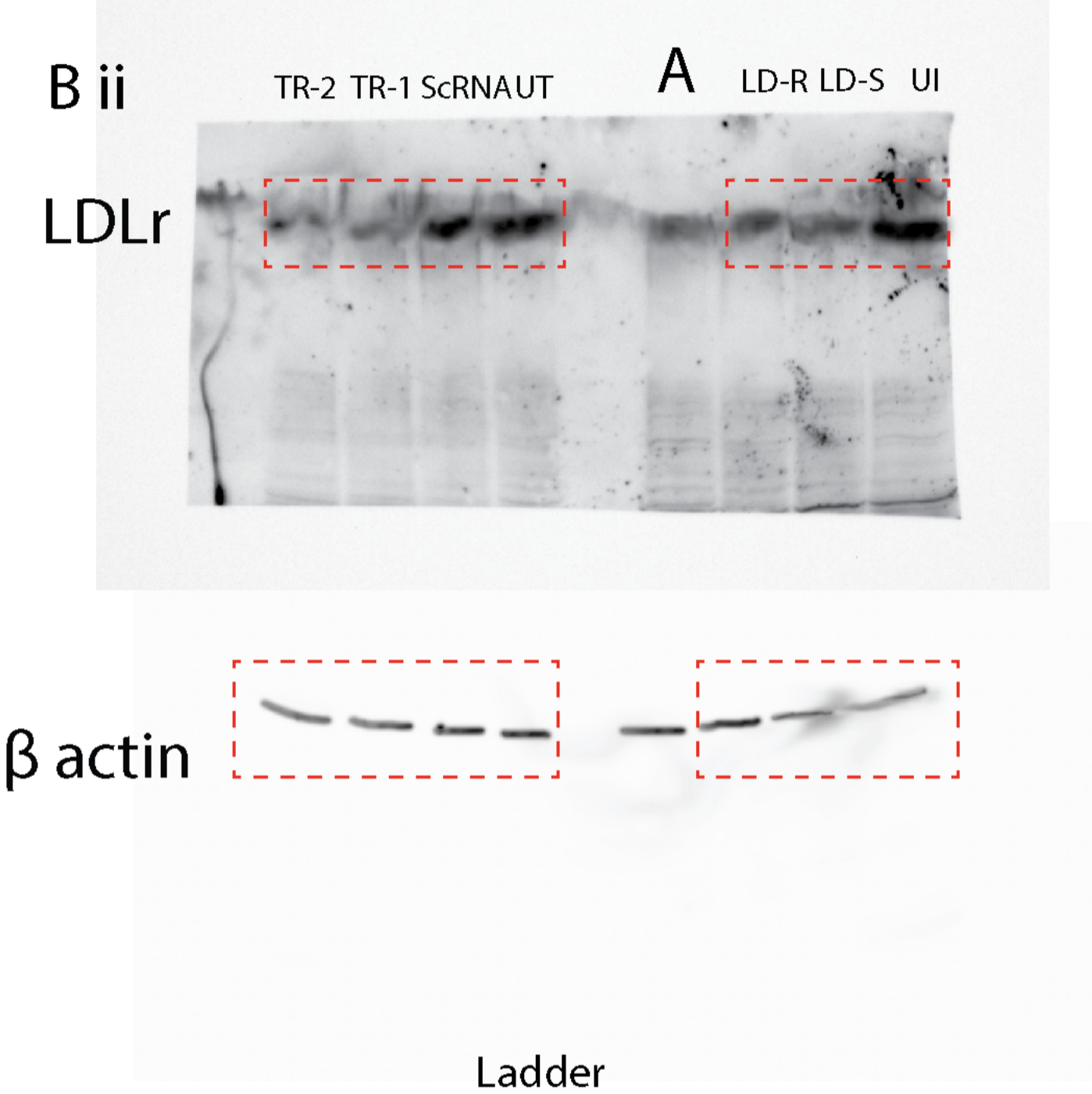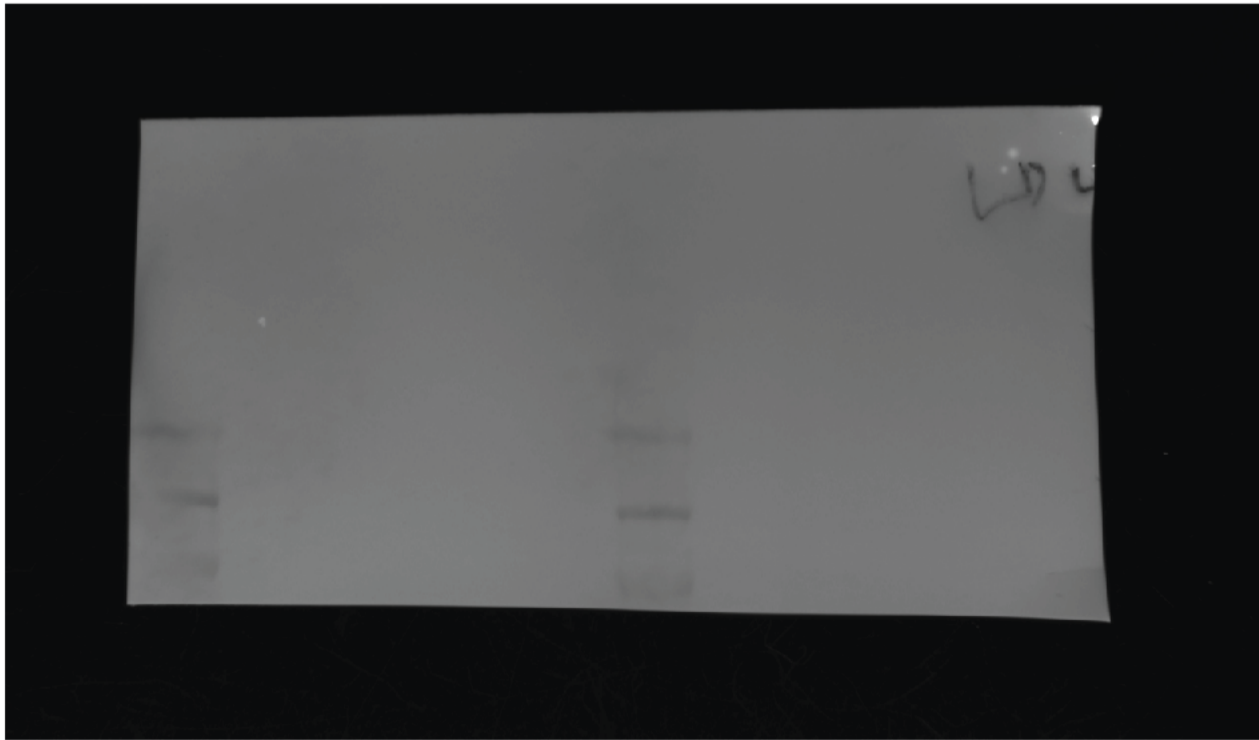

Supplement: Figure 4—figure supplement 1—source data 2. [file elife-102857-fig4-figsupp1-data2.zip › figure 4 supplementary 1 source data 1 marked/figure 4 supplementary 1 source data 1 marked.pdf]

Ci

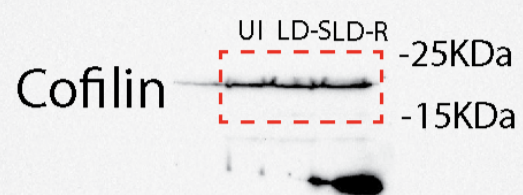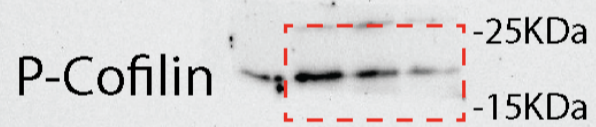

Ladder

-25KDa

-15KDa

$\beta$ -Actin

-50KDa

-37KDa

Supplement: Figure 4—figure supplement 1—source data 2. [file elife-102857-fig4-figsupp1-data2.zip › figure 4 supplementary 1 source data 1 marked/figure 4 supplementary 1 source data 2 marked.pdf]

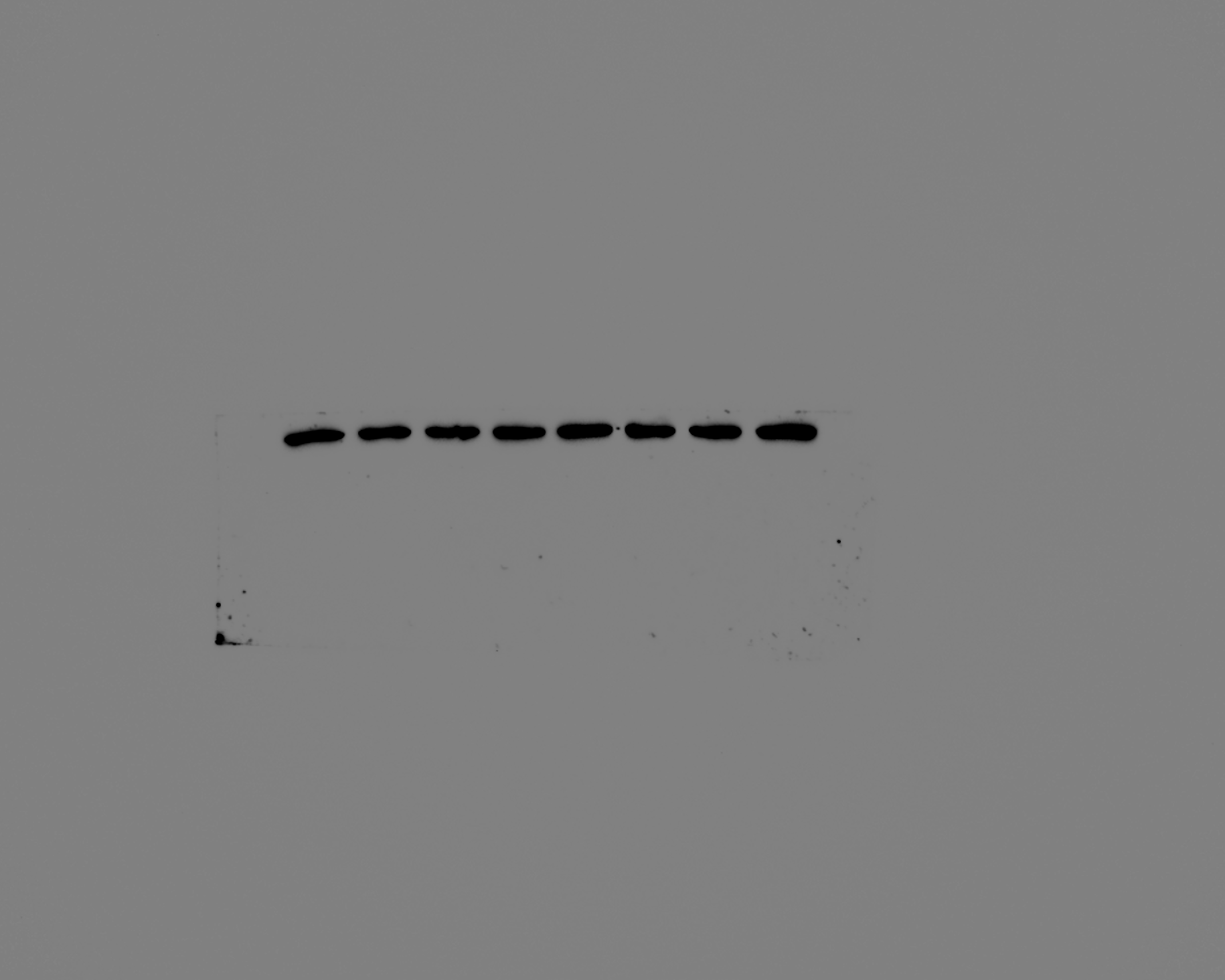

Supplement: Figure 5—source data 1. [file elife-102857-fig5-data1.zip › Figure 5 Source data 1/b actin.tif]

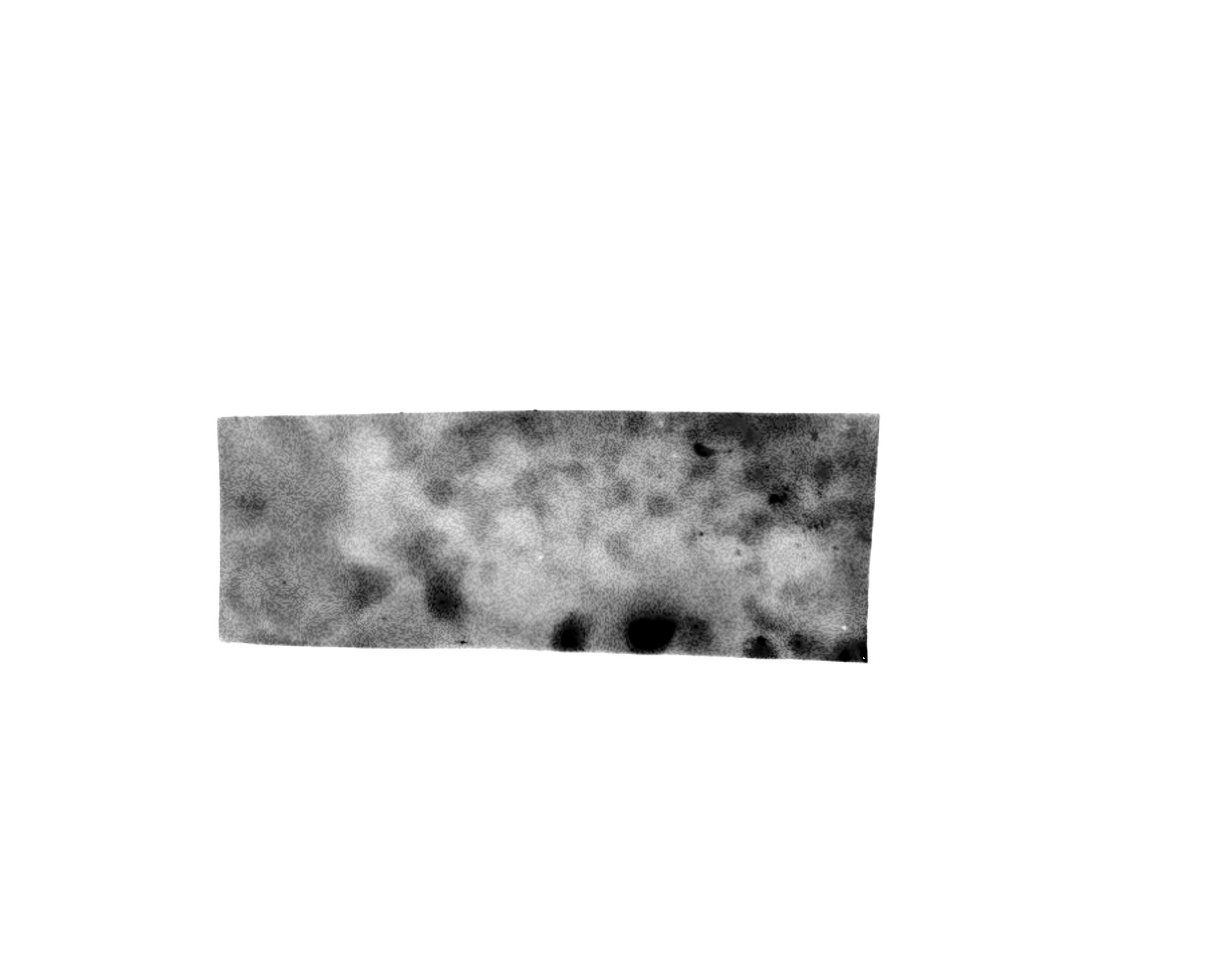

Supplement: Figure 5—source data 1. [file elife-102857-fig5-data1.zip › Figure 5 Source data 1/Ladder.tif]

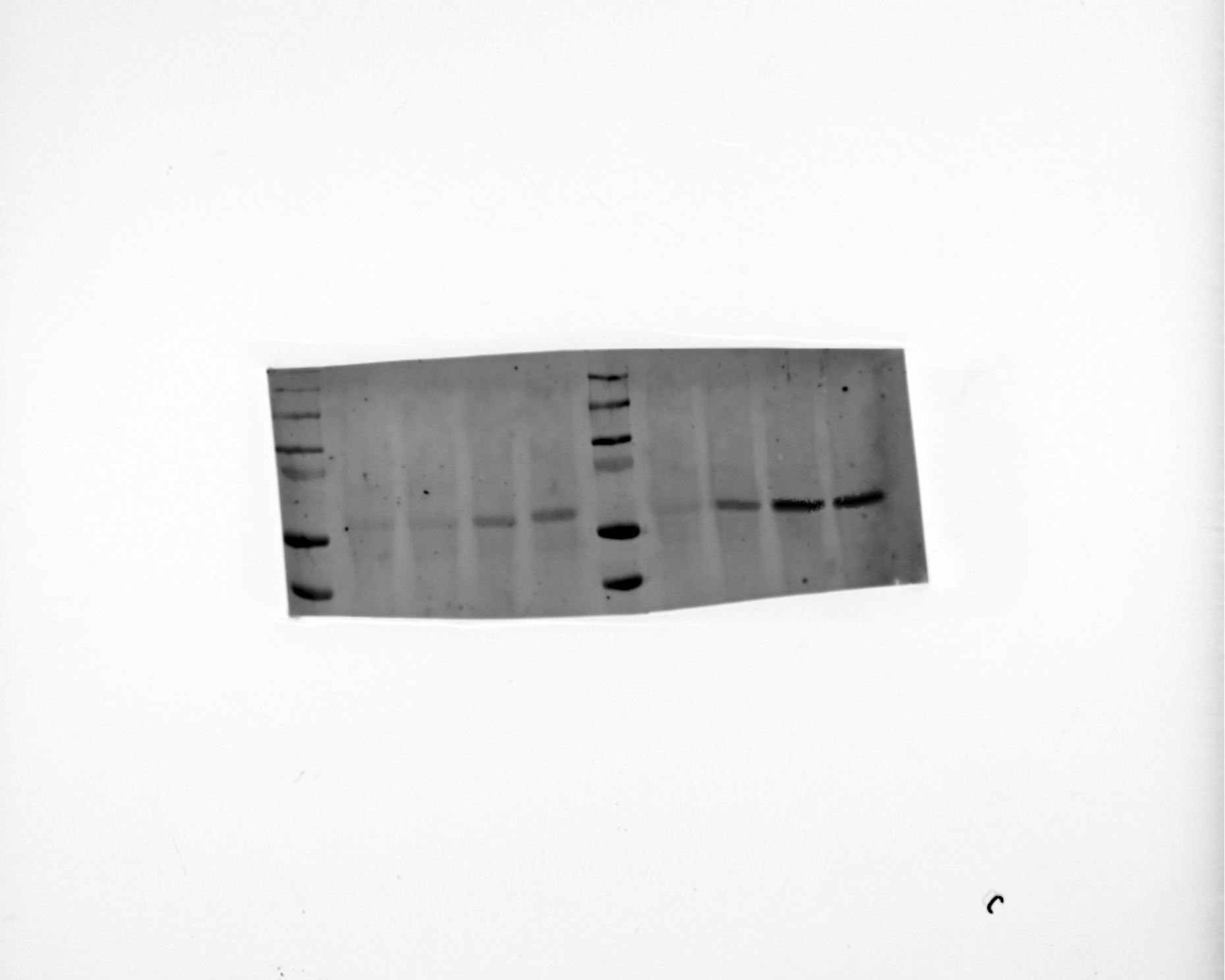

Supplement: Figure 5—source data 1. [file elife-102857-fig5-data1.zip › Figure 5 Source data 1/lamp1.tif]

Figure 5

C

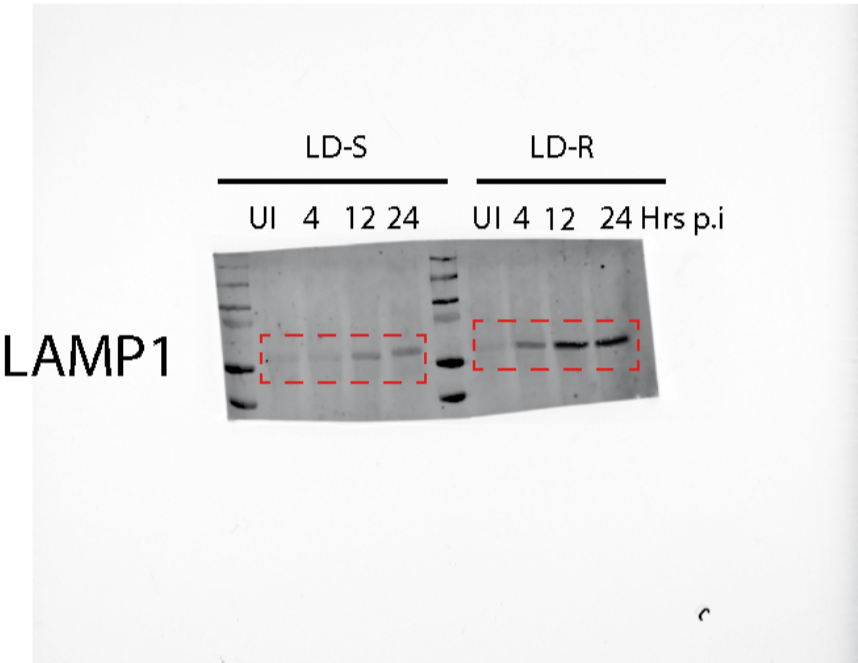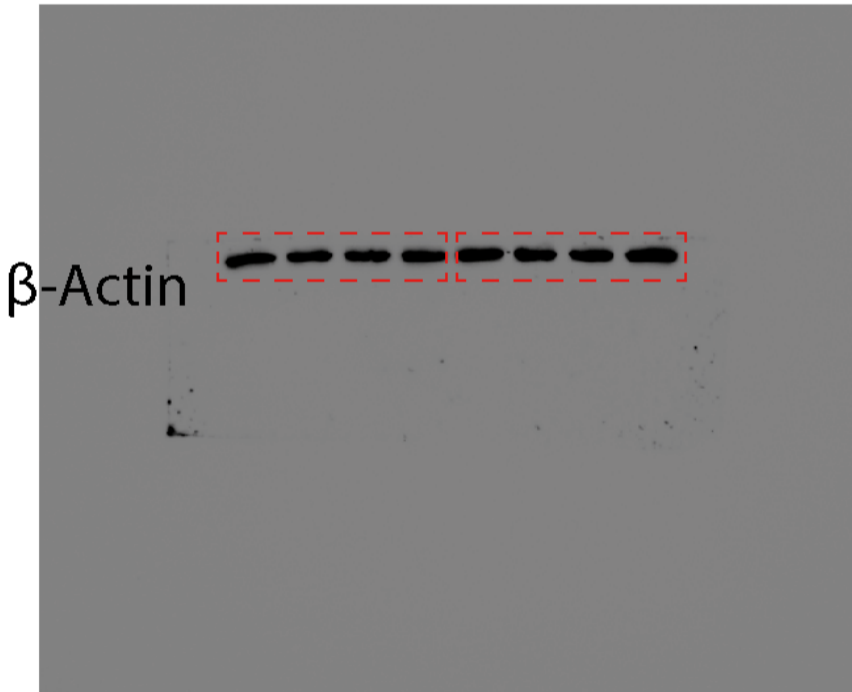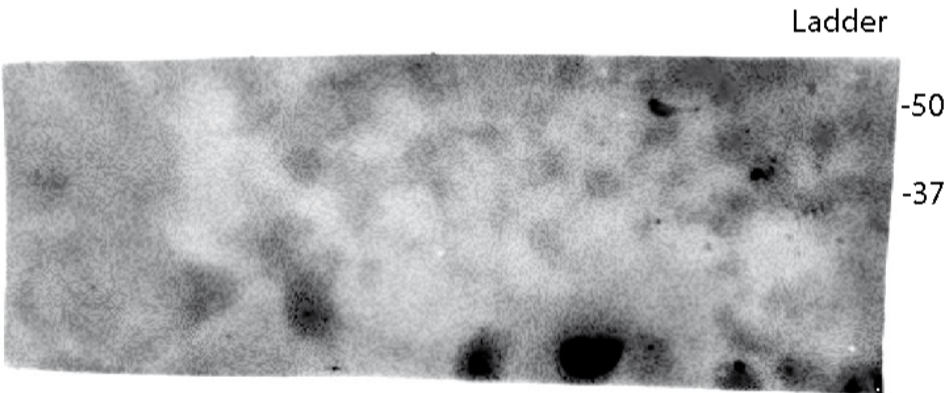

Supplement: Figure 5—source data 2. [file elife-102857-fig5-data2.zip › Figure 5 source data 1 marked/Figure 5 source data 1 marked.pdf]

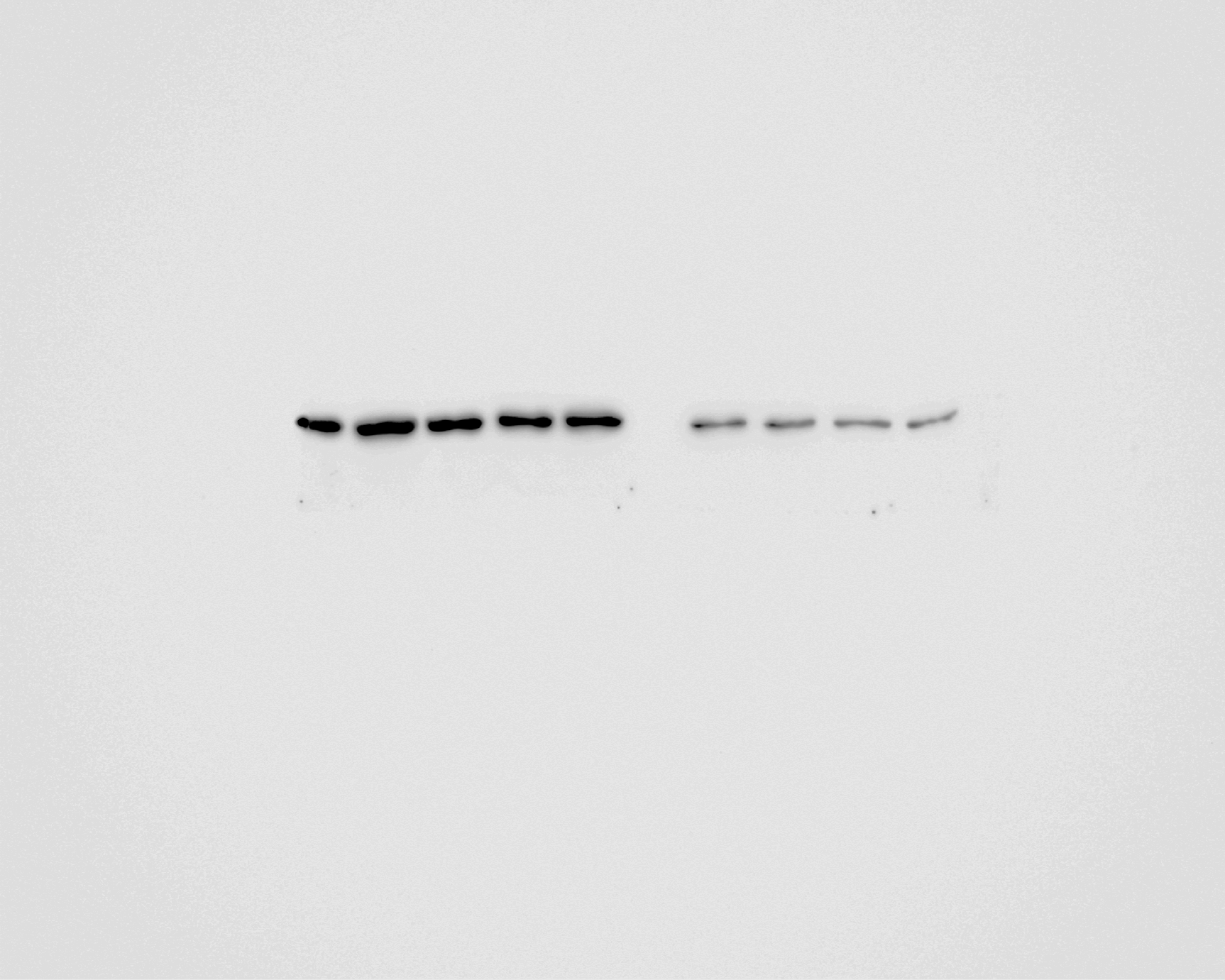

Supplement: Figure 6—source data 1. [file elife-102857-fig6-data1.zip › Figure 6 source data/Figure 6 source data 1/b actin.tif]

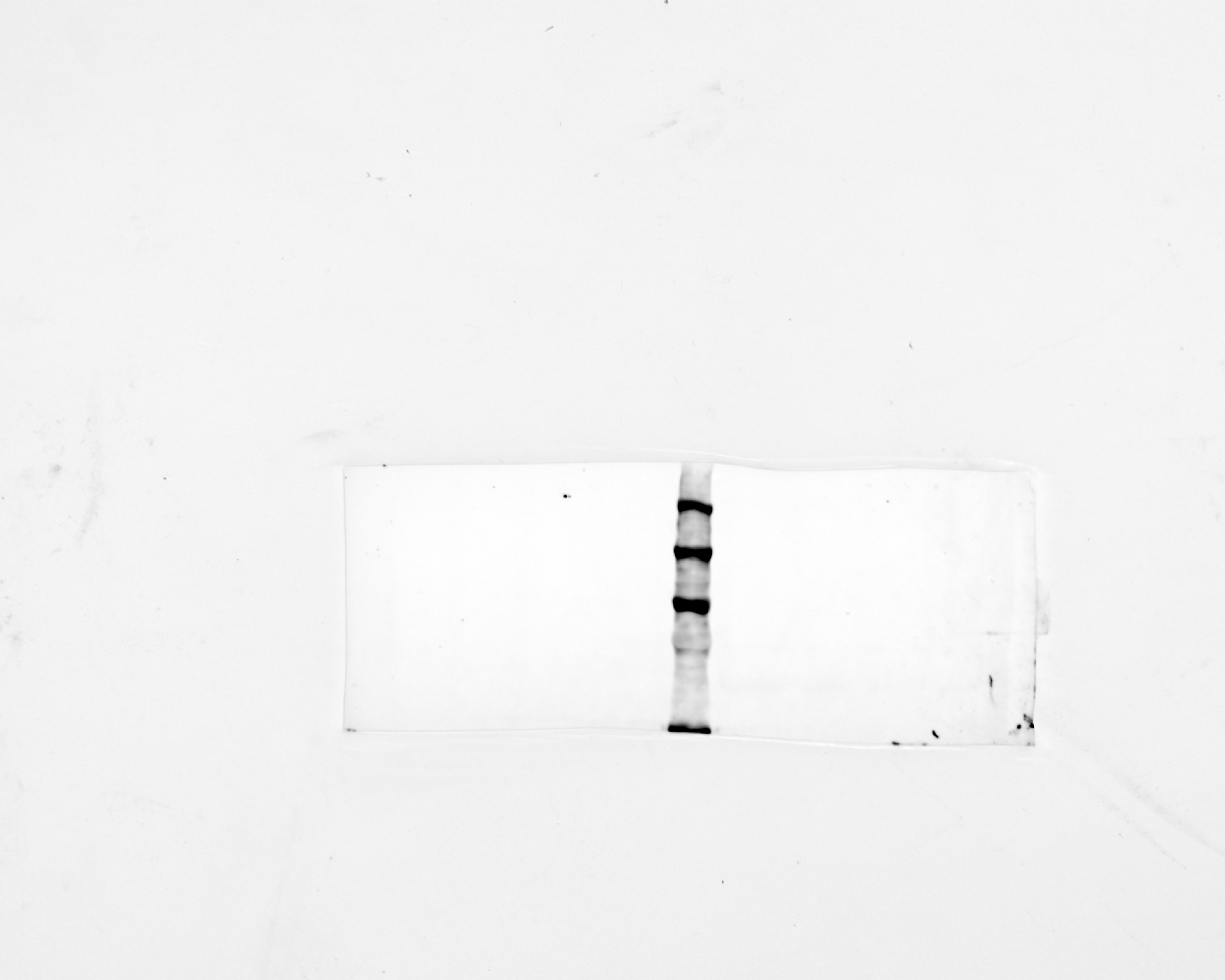

Supplement: Figure 6—source data 1. [file elife-102857-fig6-data1.zip › Figure 6 source data/Figure 6 source data 1/ladder (2).tif]

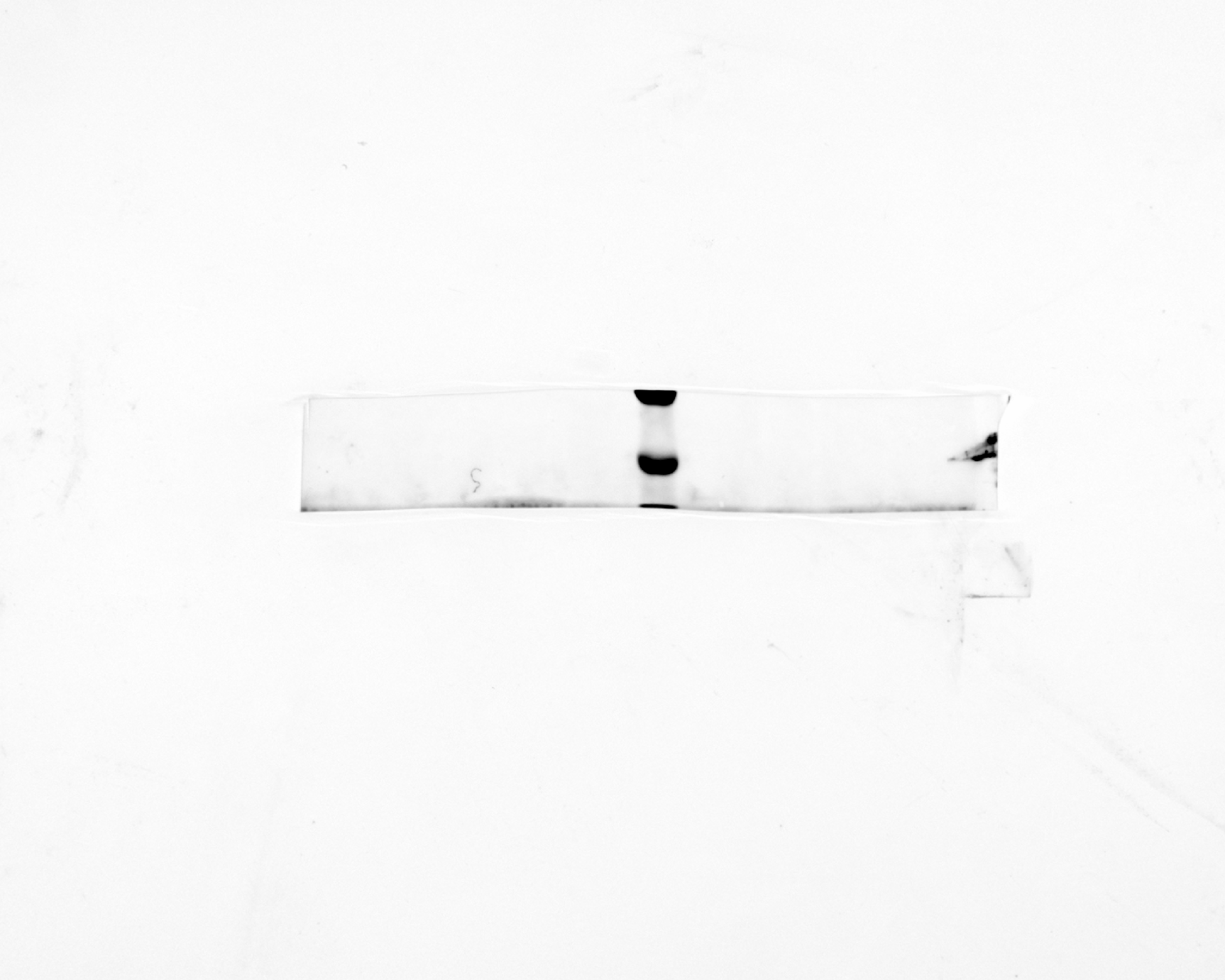

Supplement: Figure 6—source data 1. [file elife-102857-fig6-data1.zip › Figure 6 source data/Figure 6 source data 1/ladder.tif]

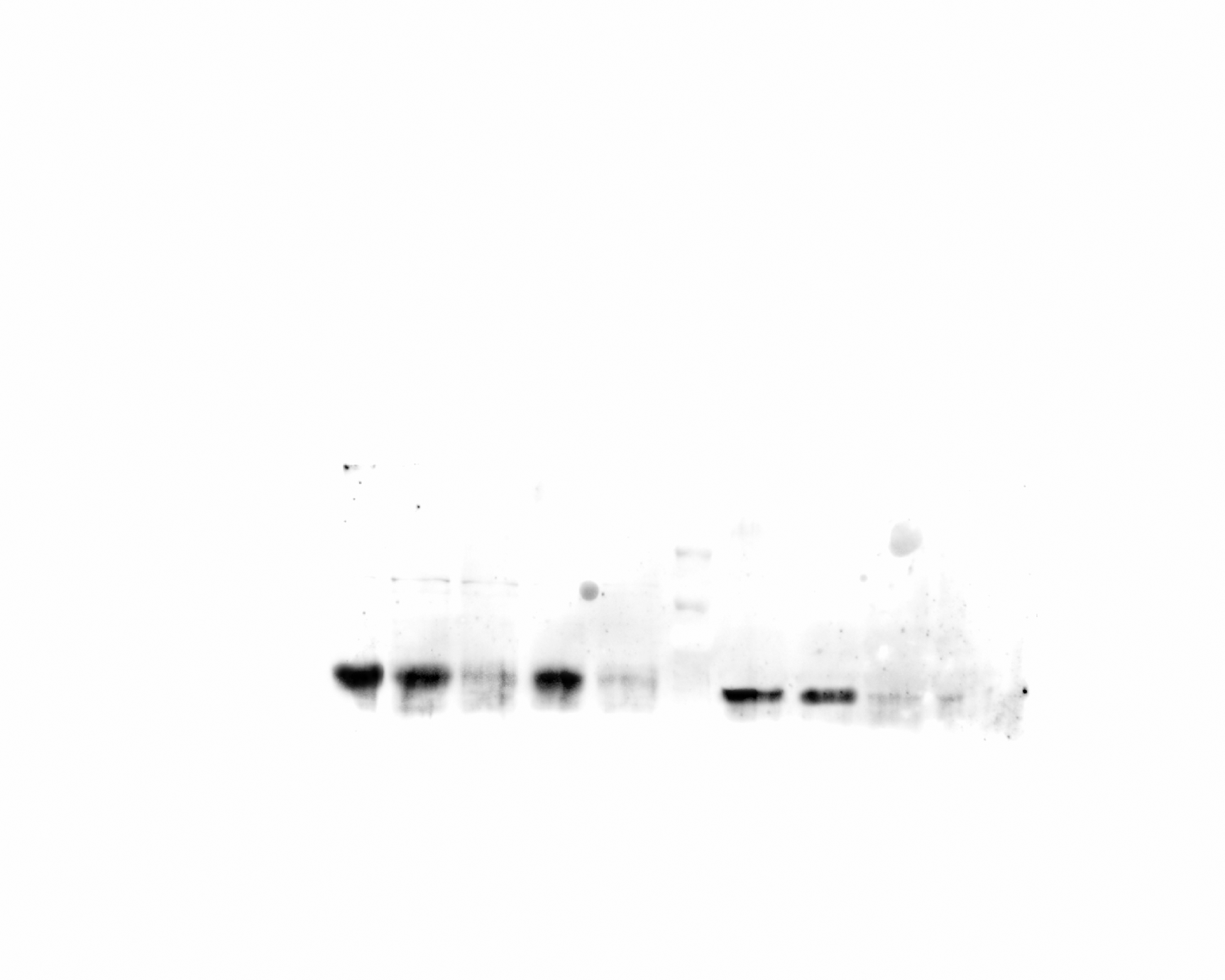

Supplement: Figure 6—source data 1. [file elife-102857-fig6-data1.zip › Figure 6 source data/Figure 6 source data 1/srebp2.tif]

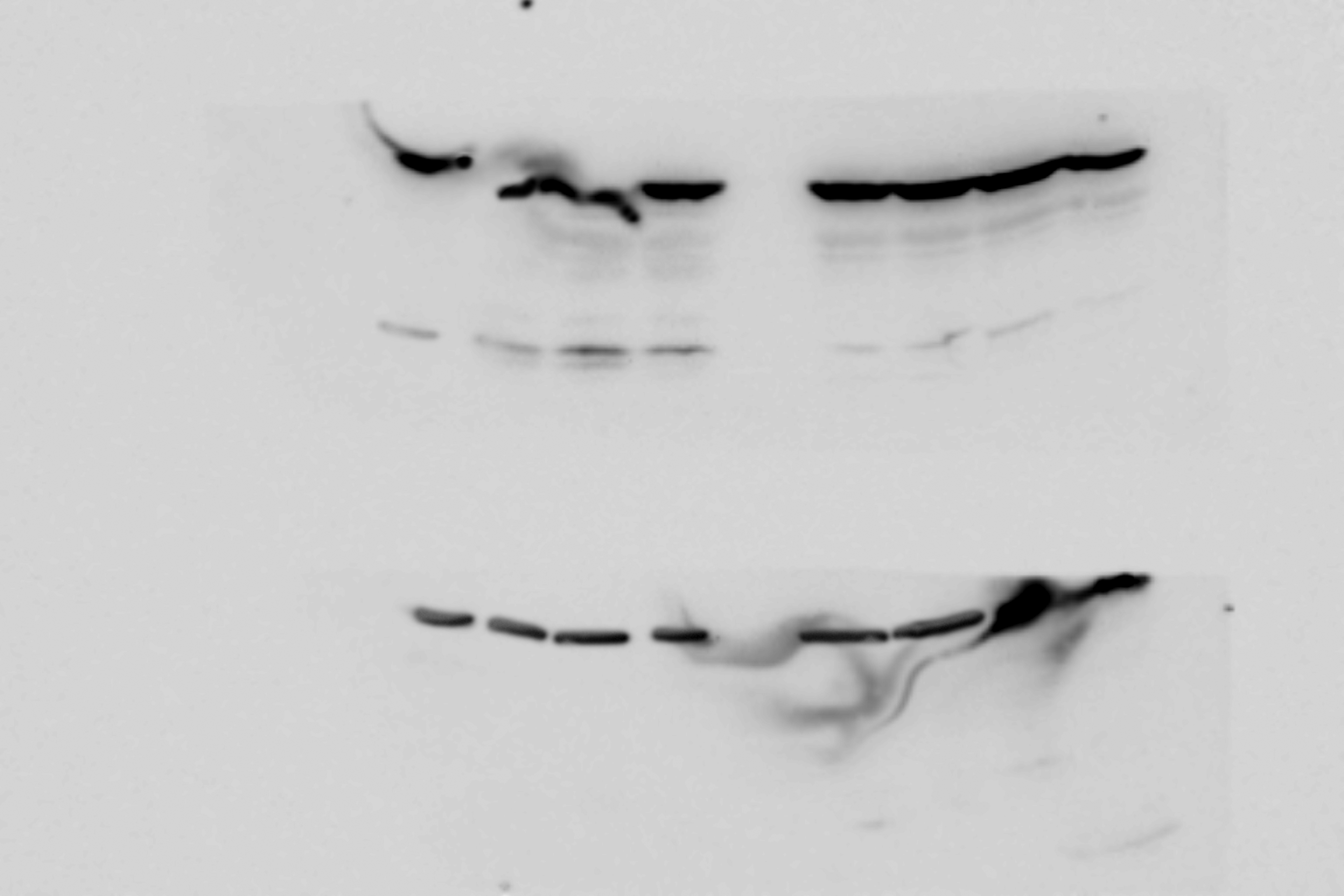

Supplement: Figure 6—source data 1. [file elife-102857-fig6-data1.zip › Figure 6 source data/Figure 6 source data 2 and 4/b actin.tif]

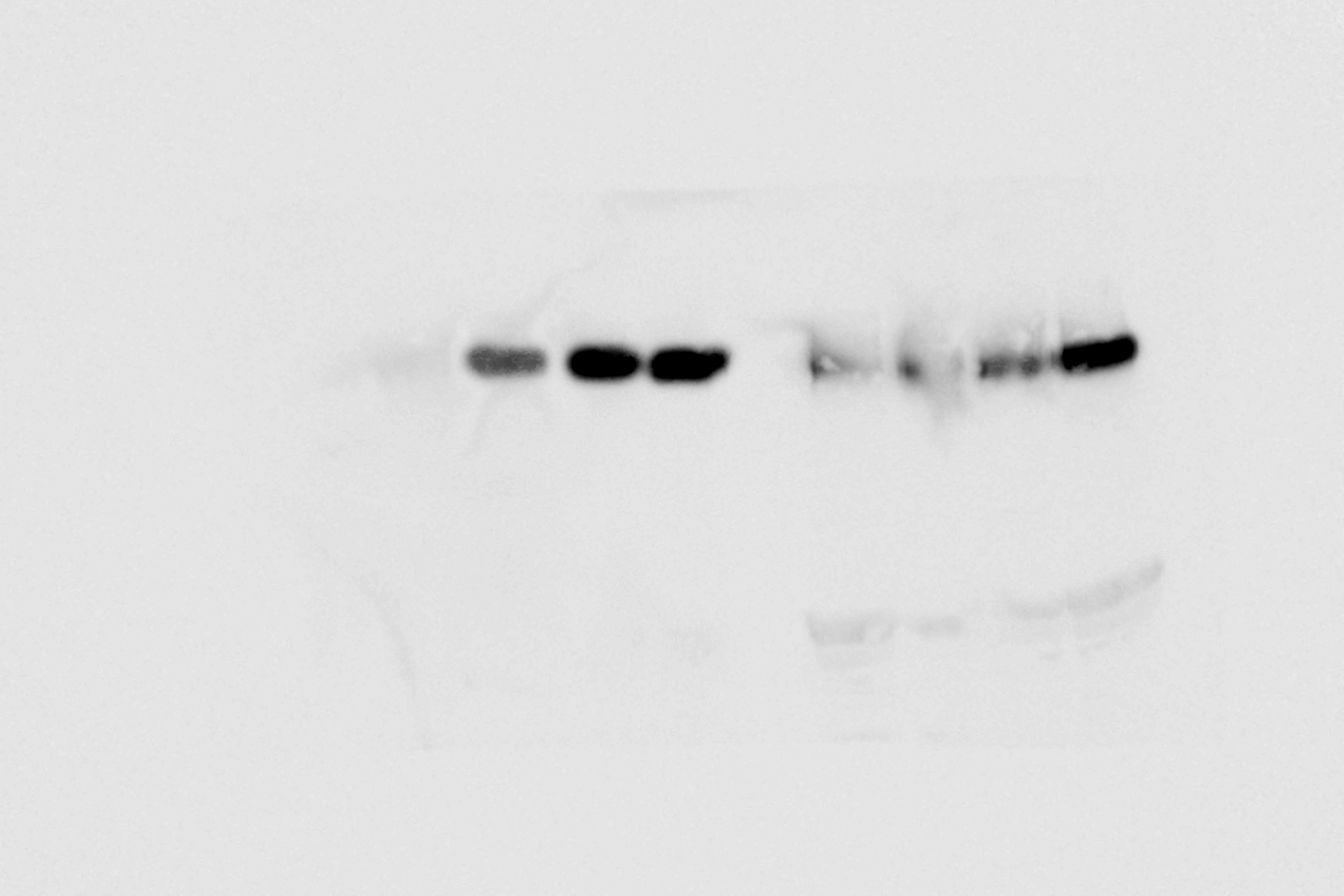

Supplement: Figure 6—source data 1. [file elife-102857-fig6-data1.zip › Figure 6 source data/Figure 6 source data 2 and 4/Final NPC-1 infection and siRNA.tif]

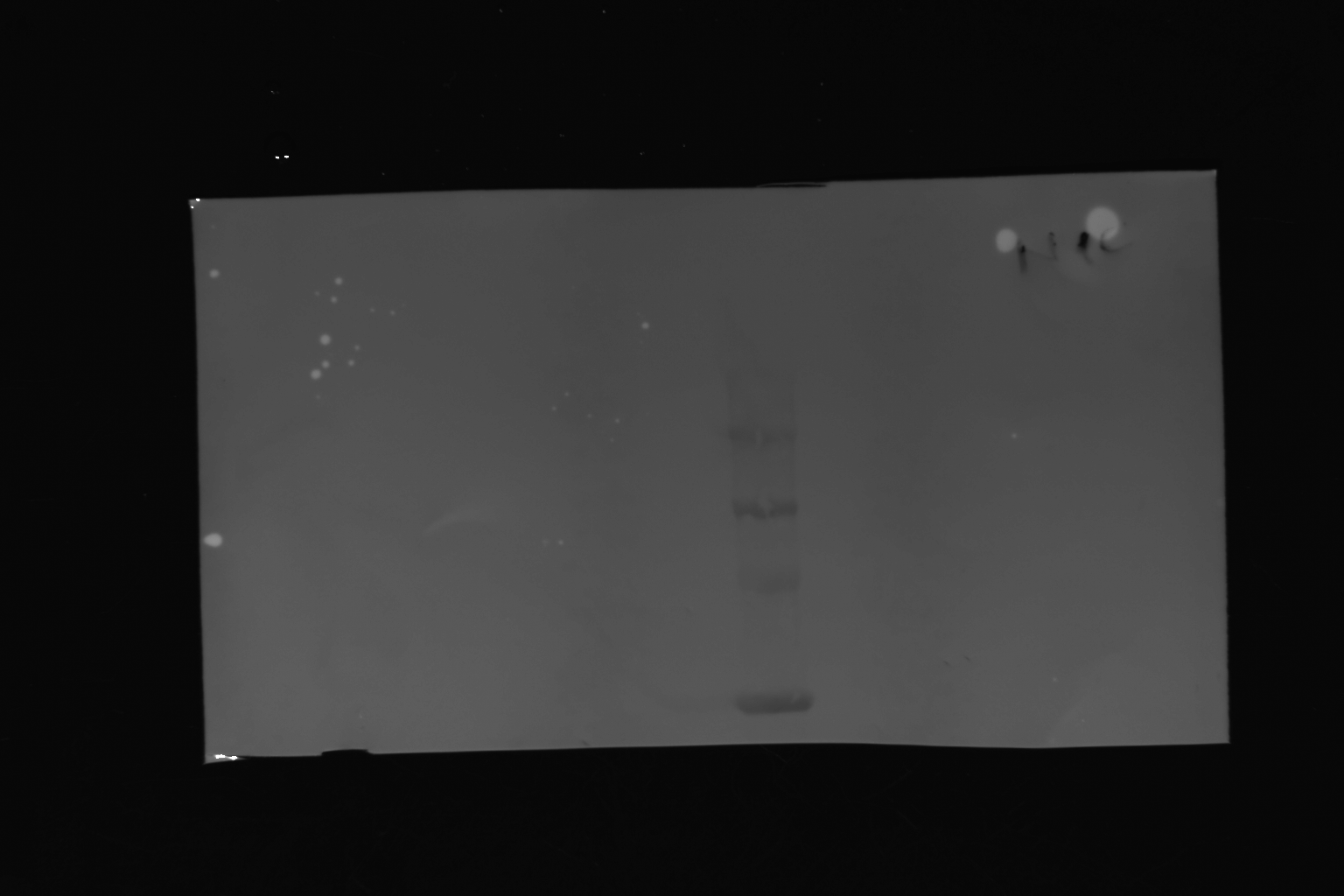

Supplement: Figure 6—source data 1. [file elife-102857-fig6-data1.zip › Figure 6 source data/Figure 6 source data 2 and 4/ladder.tif]

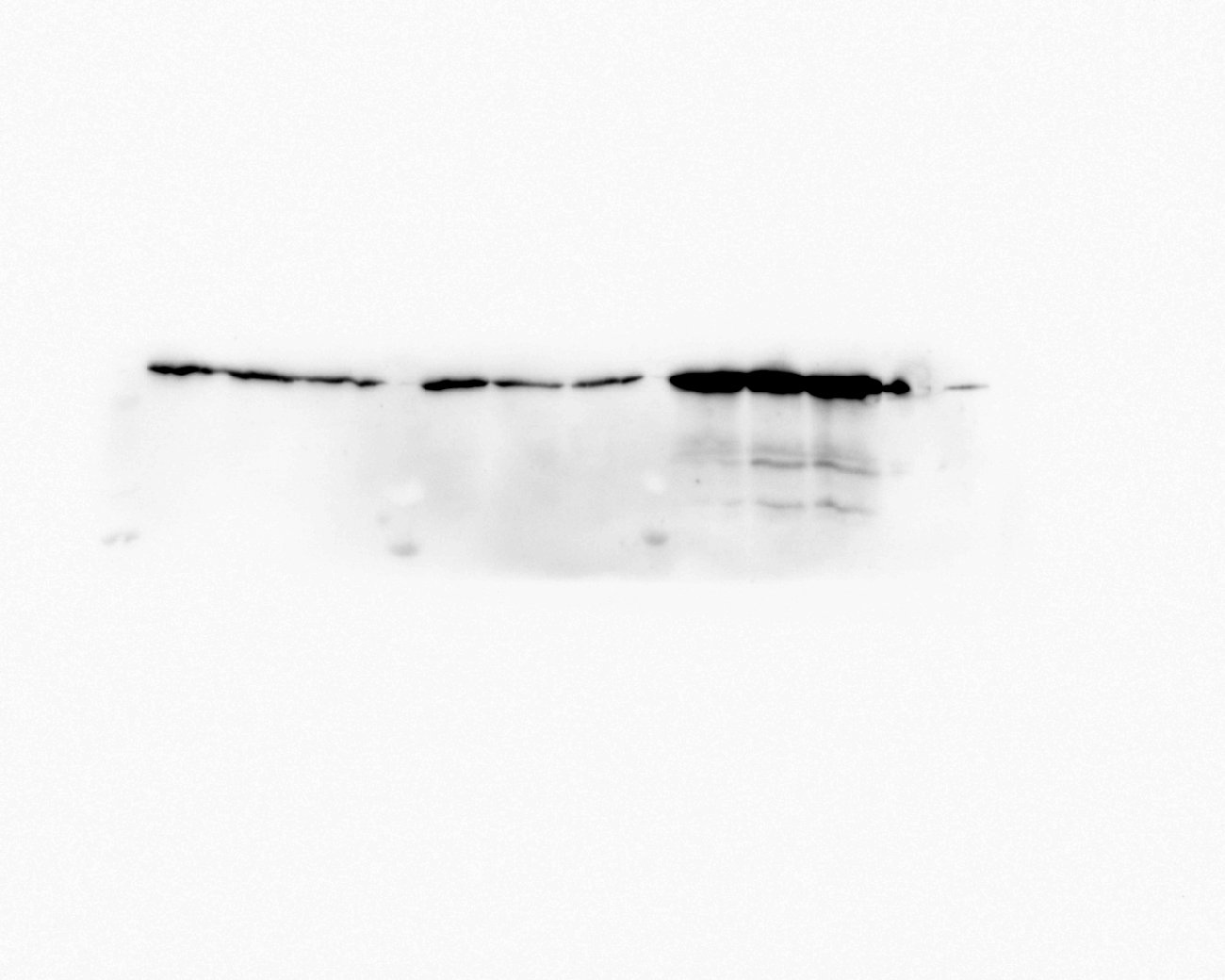

Supplement: Figure 6—source data 1. [file elife-102857-fig6-data1.zip › Figure 6 source data/Figure 6 source data 3/b actin.tif]

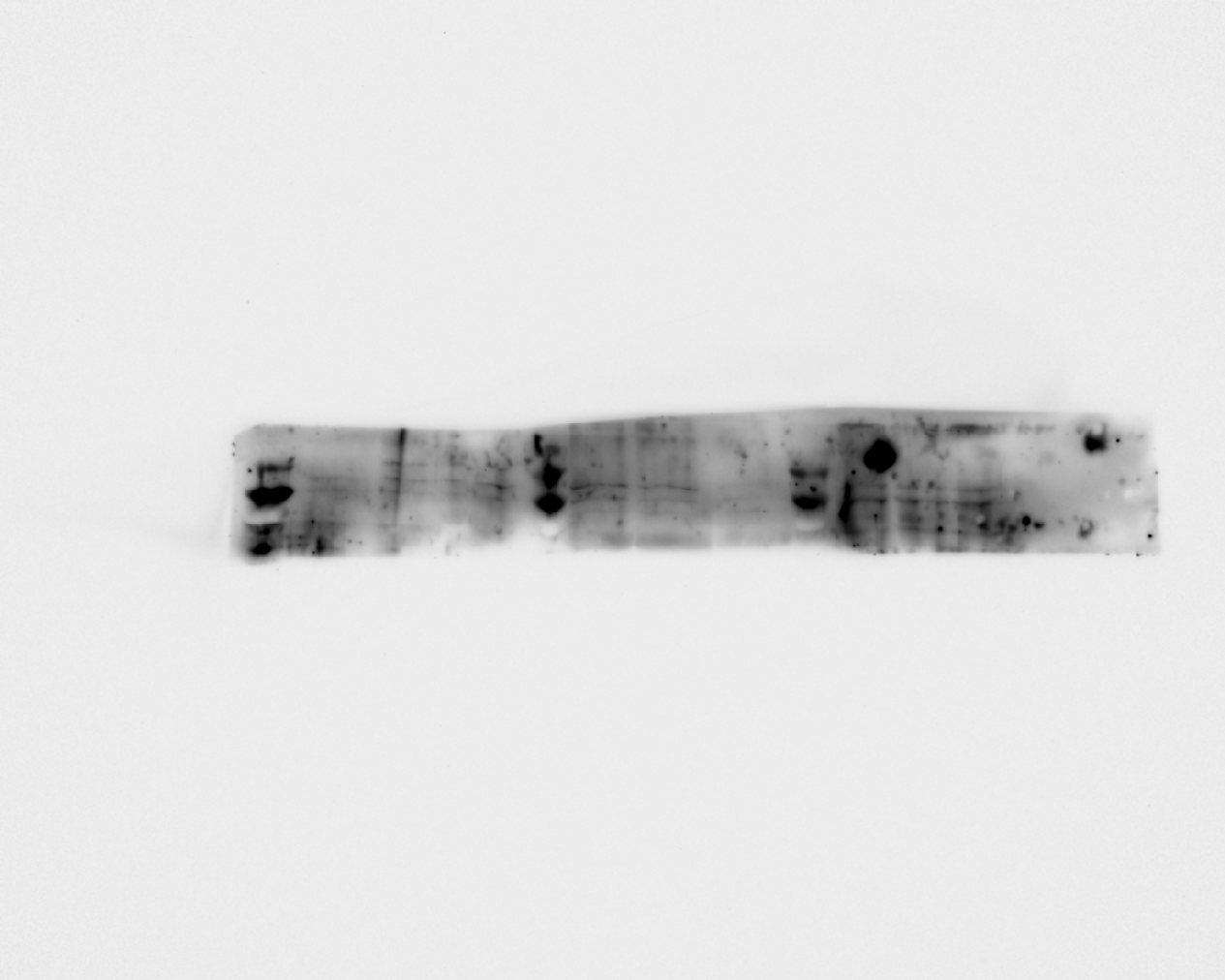

Supplement: Figure 6—source data 1. [file elife-102857-fig6-data1.zip › Figure 6 source data/Figure 6 source data 3/hmgcr.tif]

Figure 6

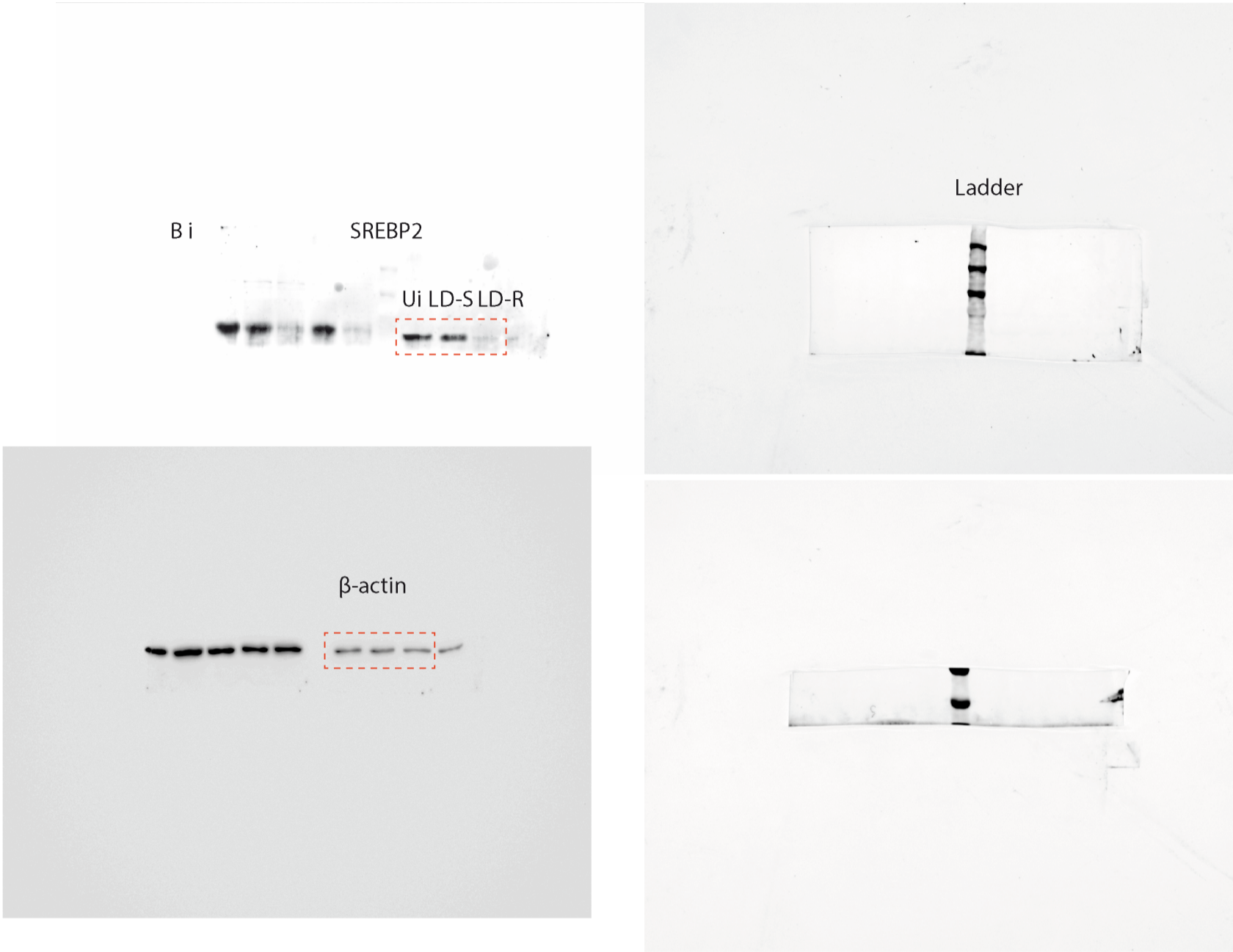

Supplement: Figure 6—source data 2. [file elife-102857-fig6-data2.zip › Figure 6 source data marked/Figure 6 source data 1 marked.pdf]

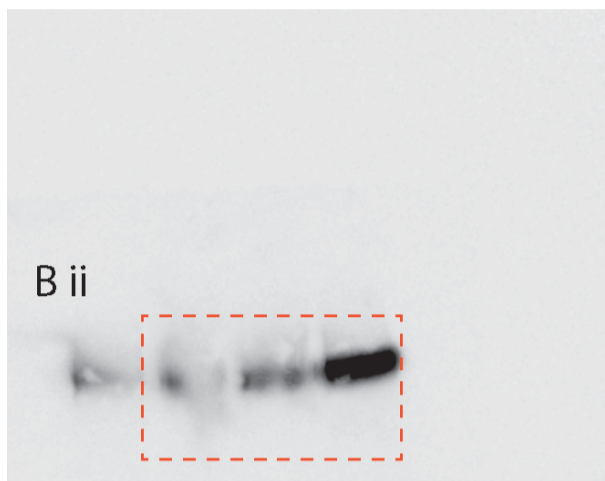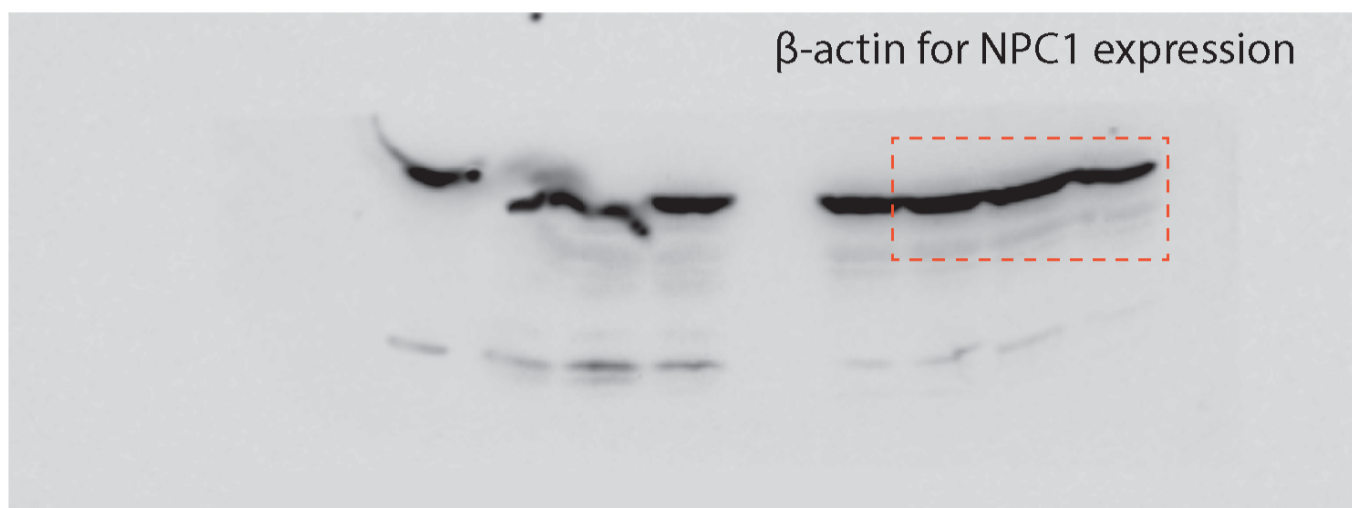

Supplement: Figure 6—source data 2. [file elife-102857-fig6-data2.zip › Figure 6 source data marked/Figure 6 source data 2 marked.pdf]

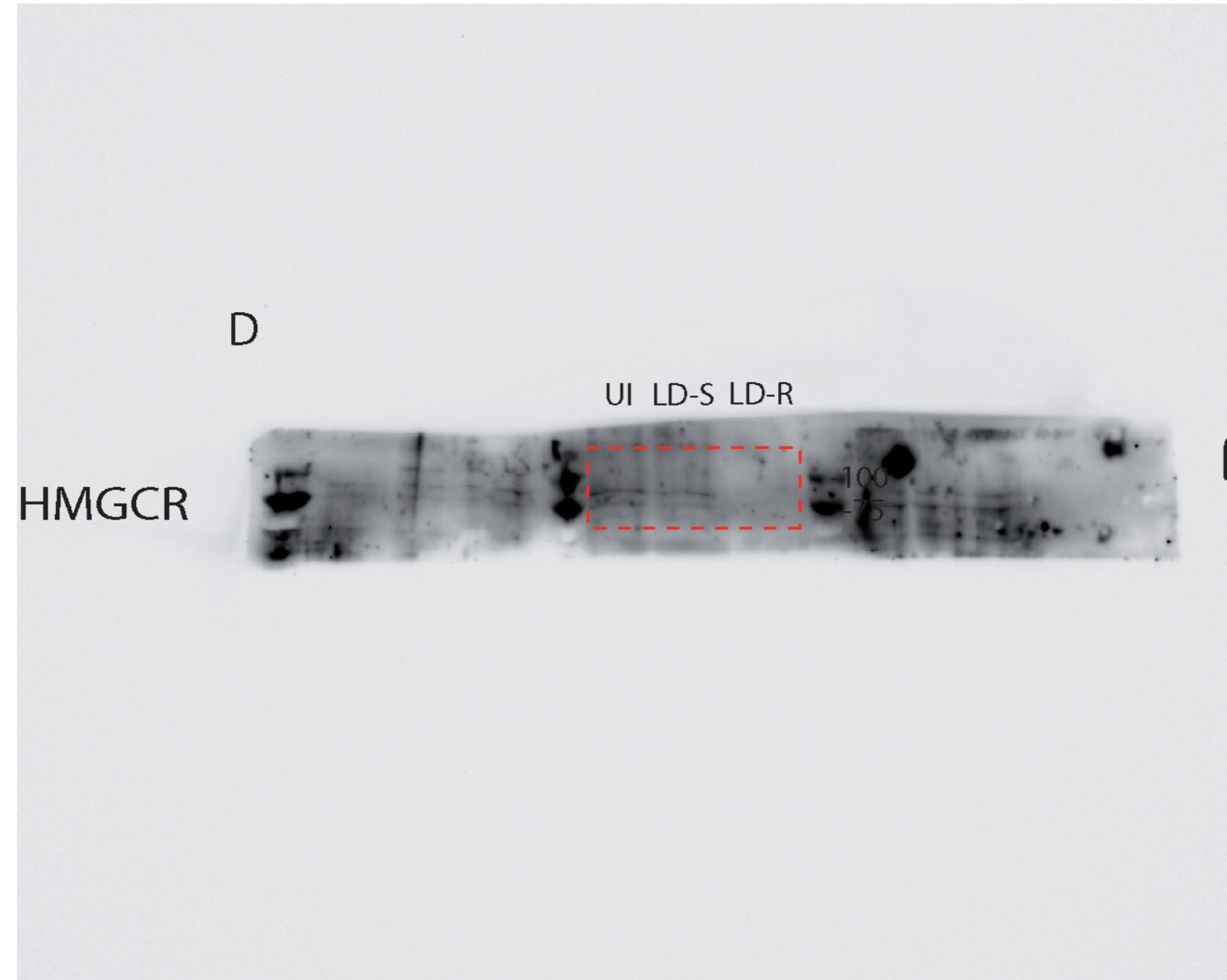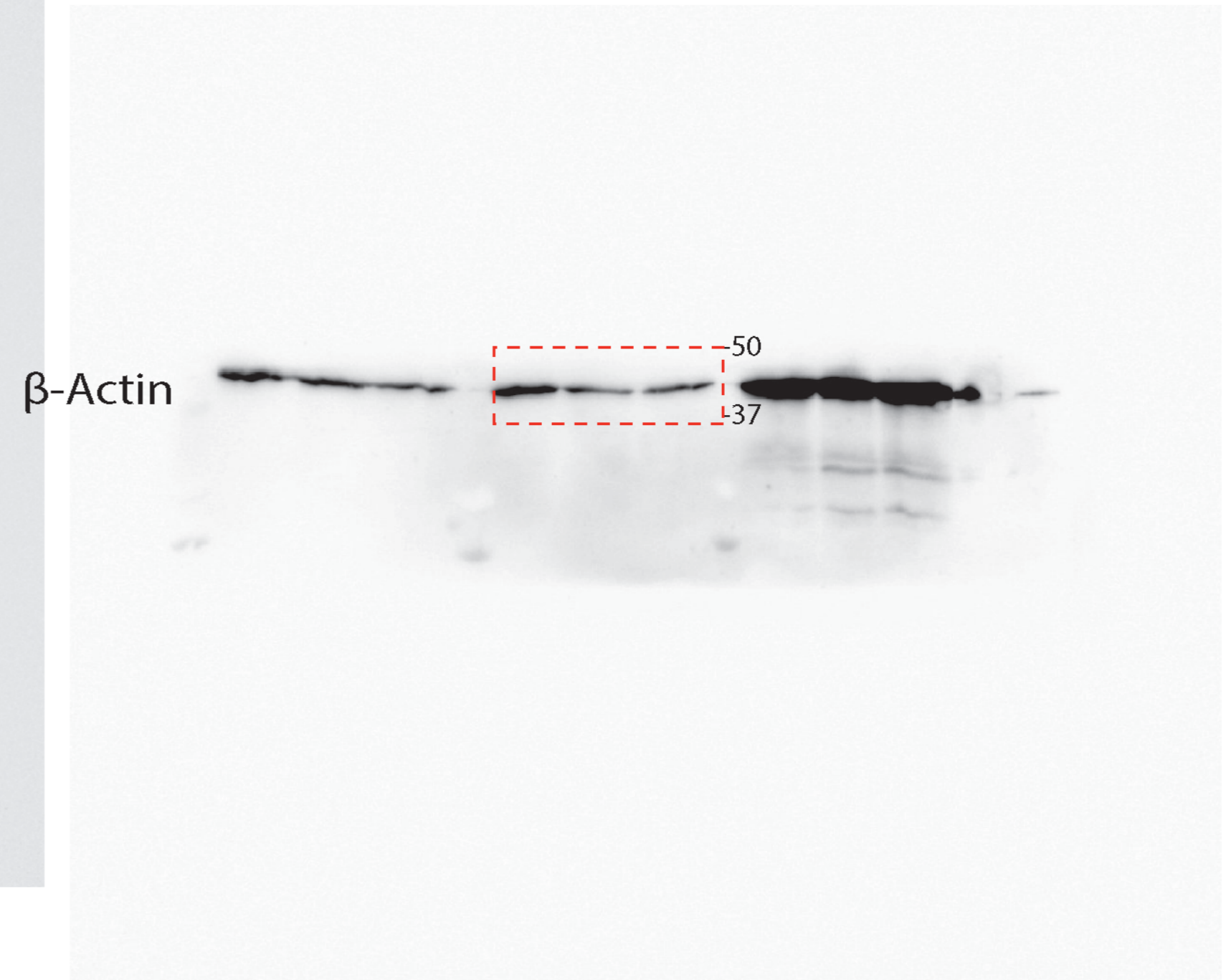

Supplement: Figure 6—source data 2. [file elife-102857-fig6-data2.zip › Figure 6 source data marked/Figure 6 source data 3 marked.pdf]

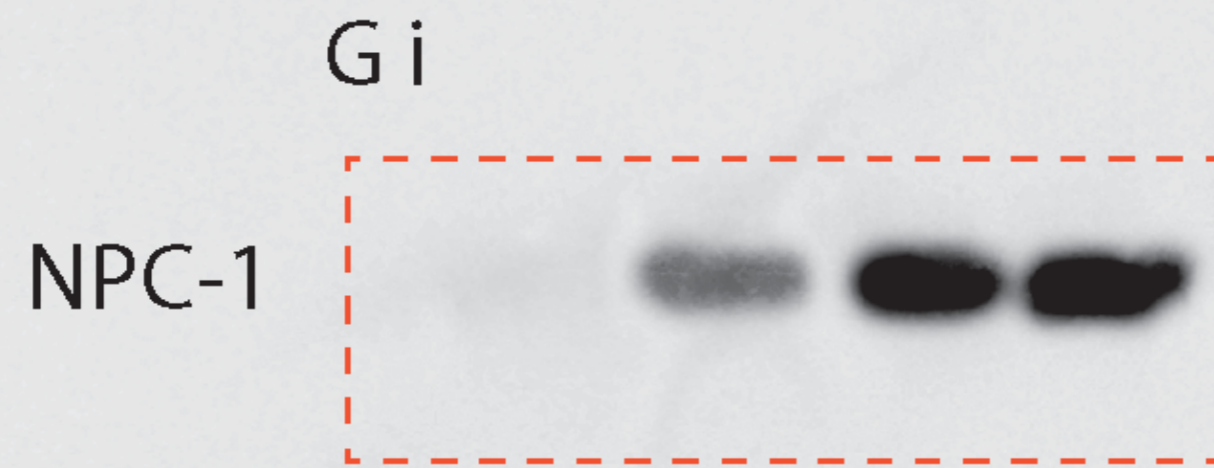

$\beta$ -actin for NPC1 knock down

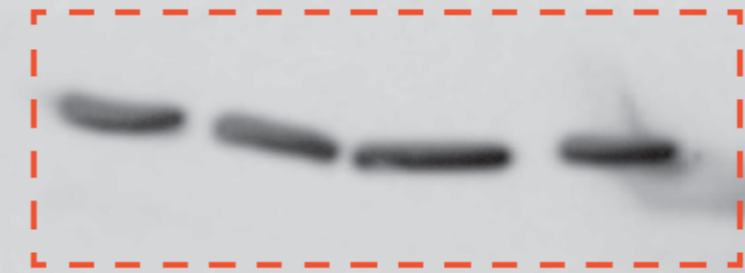

Supplement: Figure 6—source data 2. [file elife-102857-fig6-data2.zip › Figure 6 source data marked/Figure 6 source data 4 marked.pdf]

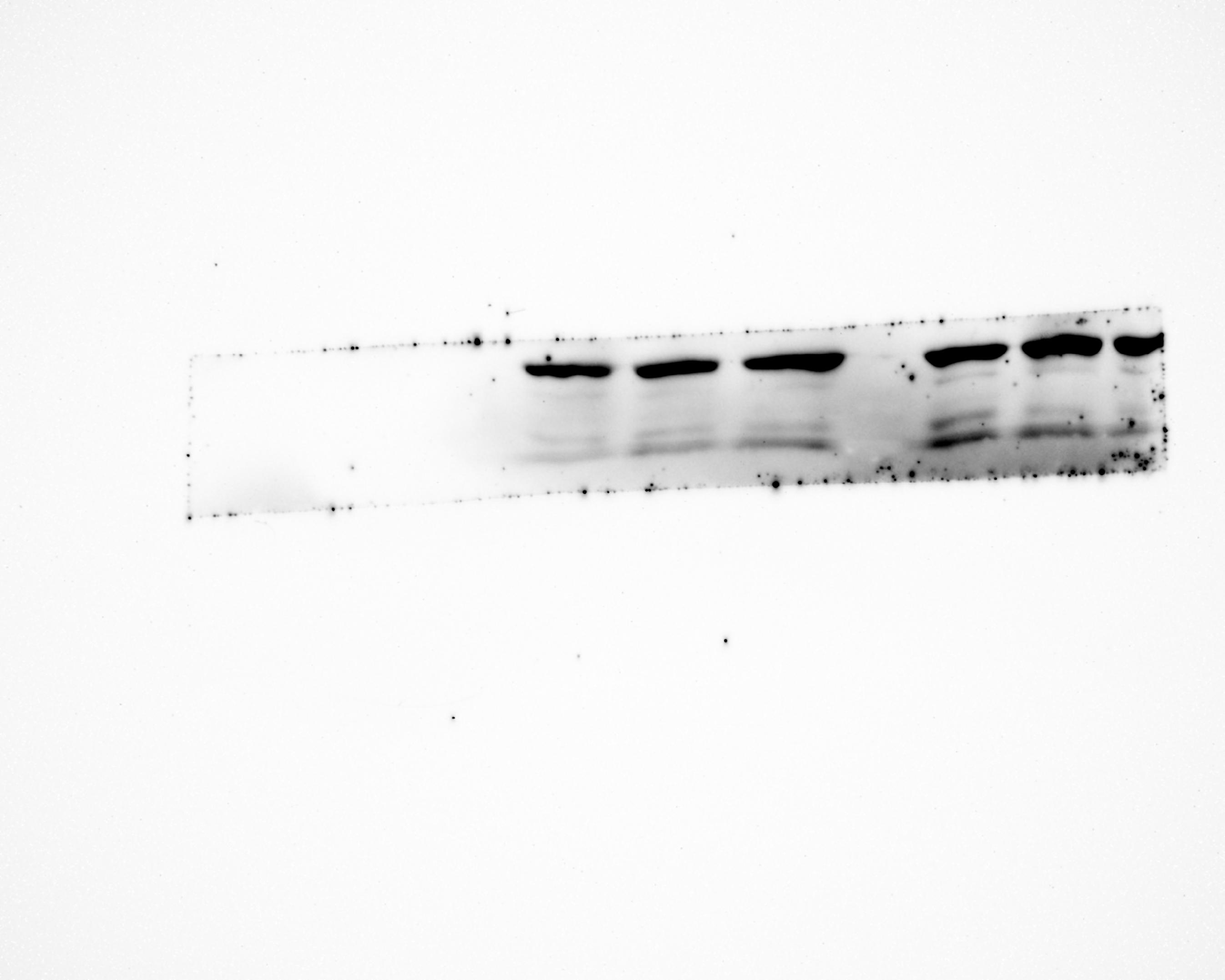

Supplement: Figure 7—source data 1. [file elife-102857-fig7-data1.zip › Figure 7 source data/msr1/b actin.tif]

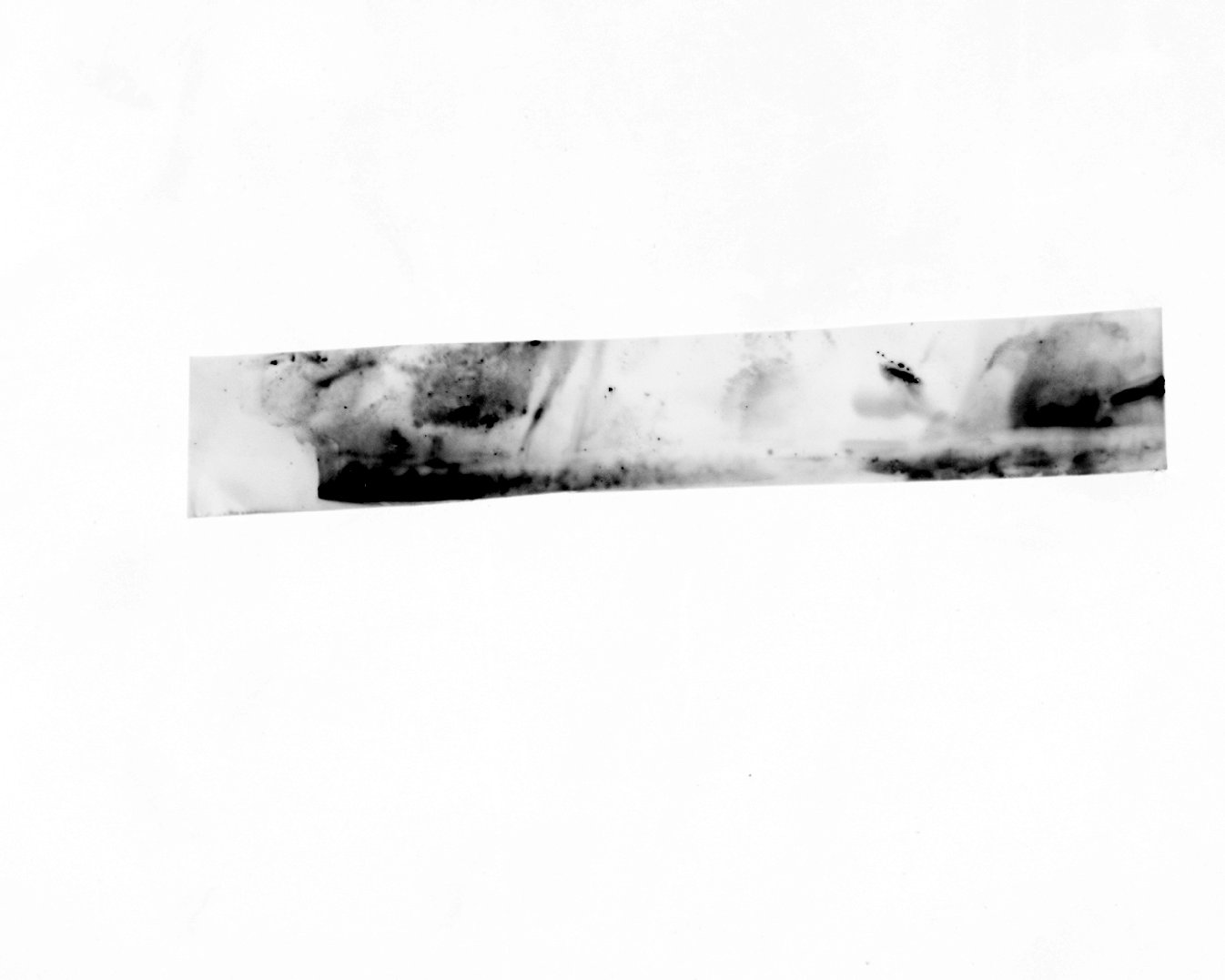

Supplement: Figure 7—source data 1. [file elife-102857-fig7-data1.zip › Figure 7 source data/msr1/ladder b actin.jpg]

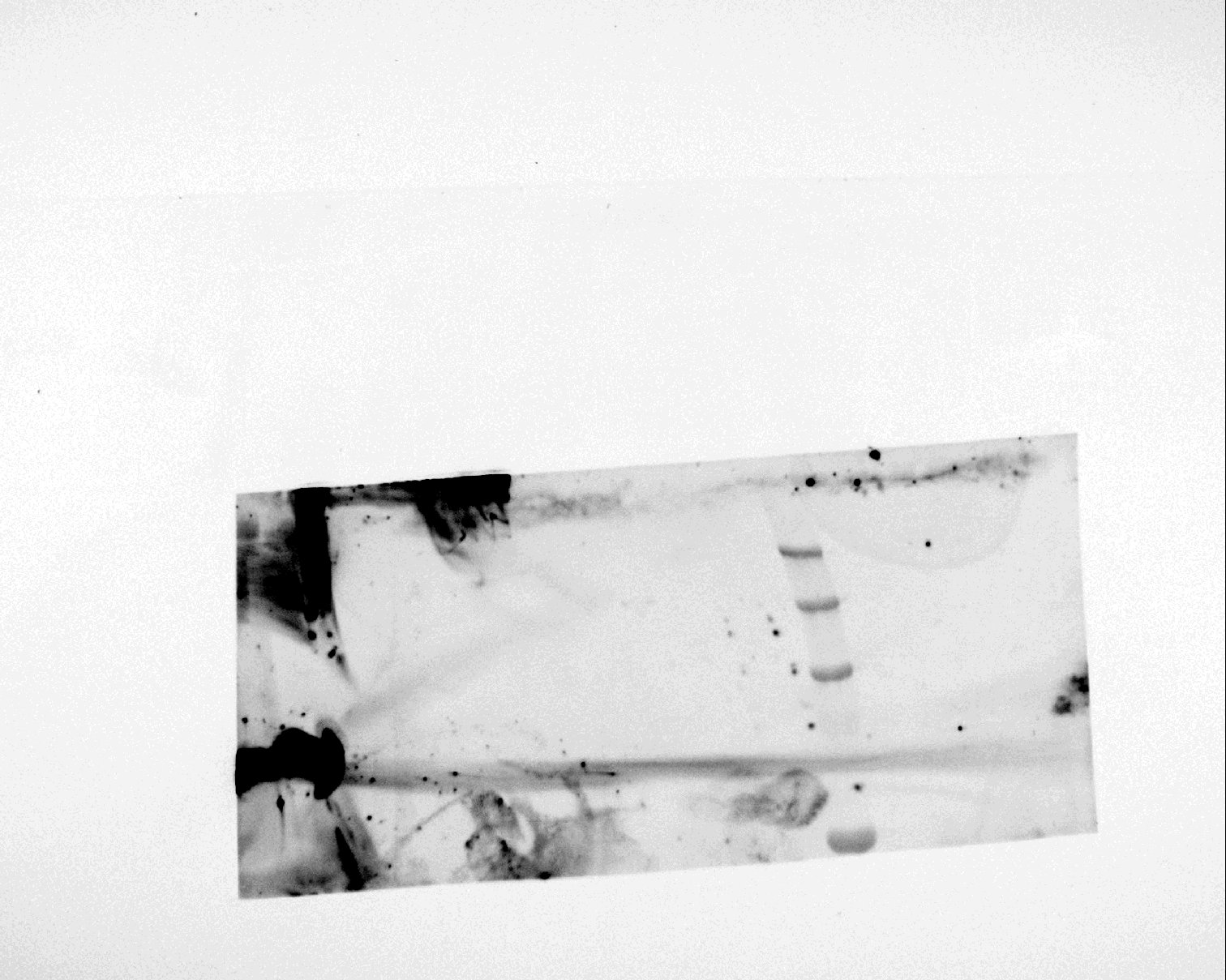

Supplement: Figure 7—source data 1. [file elife-102857-fig7-data1.zip › Figure 7 source data/msr1/ladder msr1.tif]

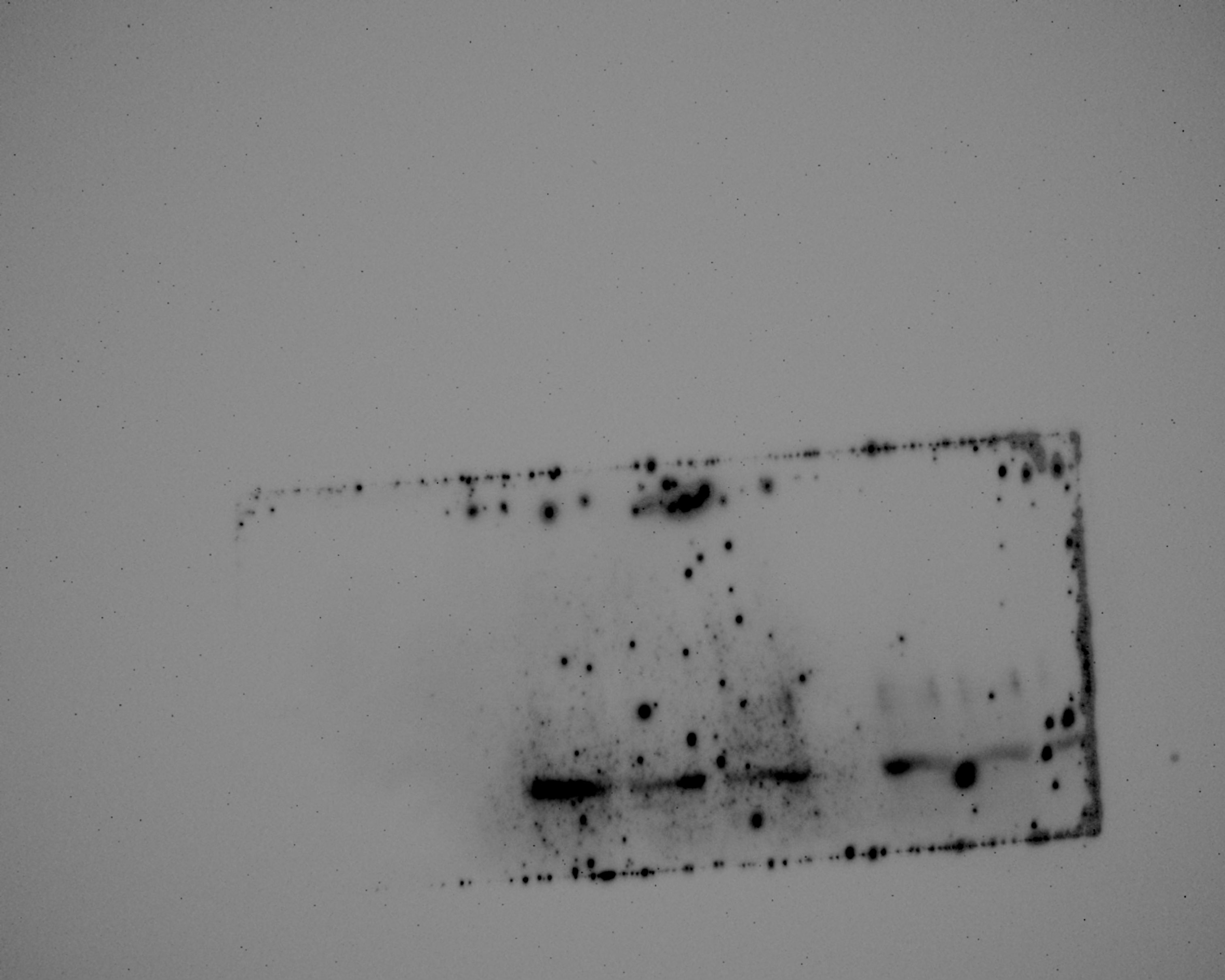

Supplement: Figure 7—source data 1. [file elife-102857-fig7-data1.zip › Figure 7 source data/msr1/msr1.tif]

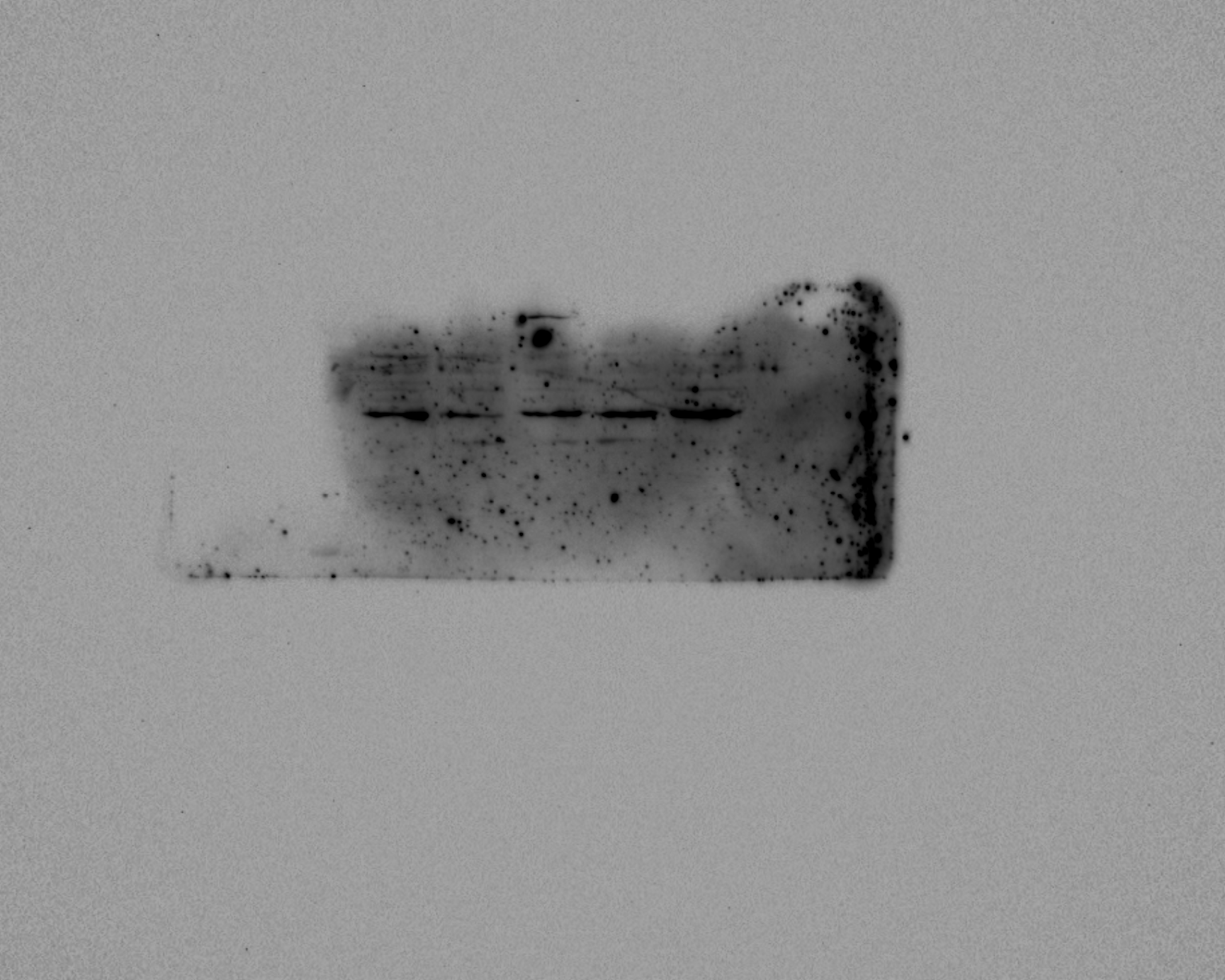

Supplement: Figure 7—source data 1. [file elife-102857-fig7-data1.zip › Figure 7 source data/VATPase/ATPV0d2.tif]

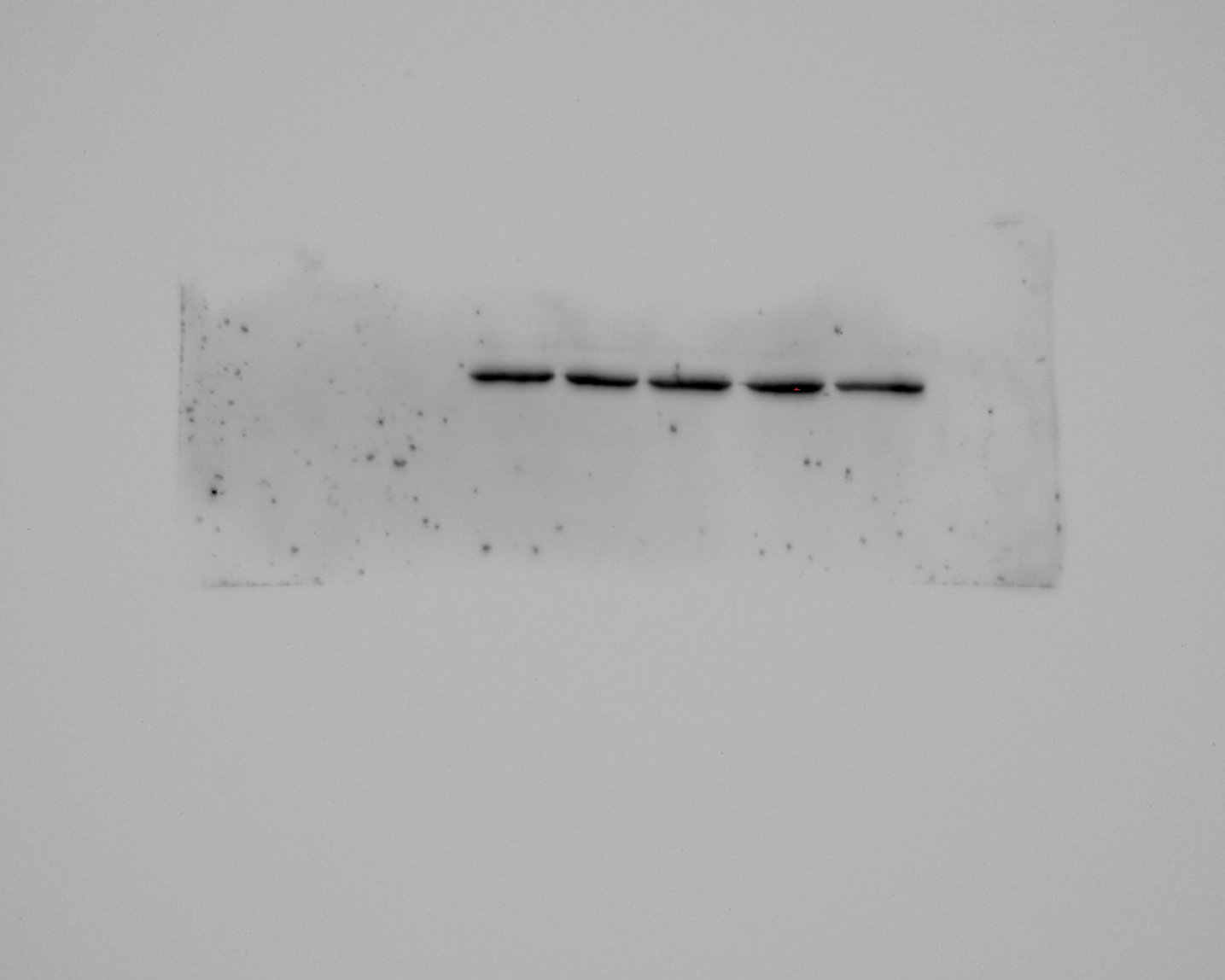

Supplement: Figure 7—source data 1. [file elife-102857-fig7-data1.zip › Figure 7 source data/VATPase/B actin.jpg]

Figure 7

D ii

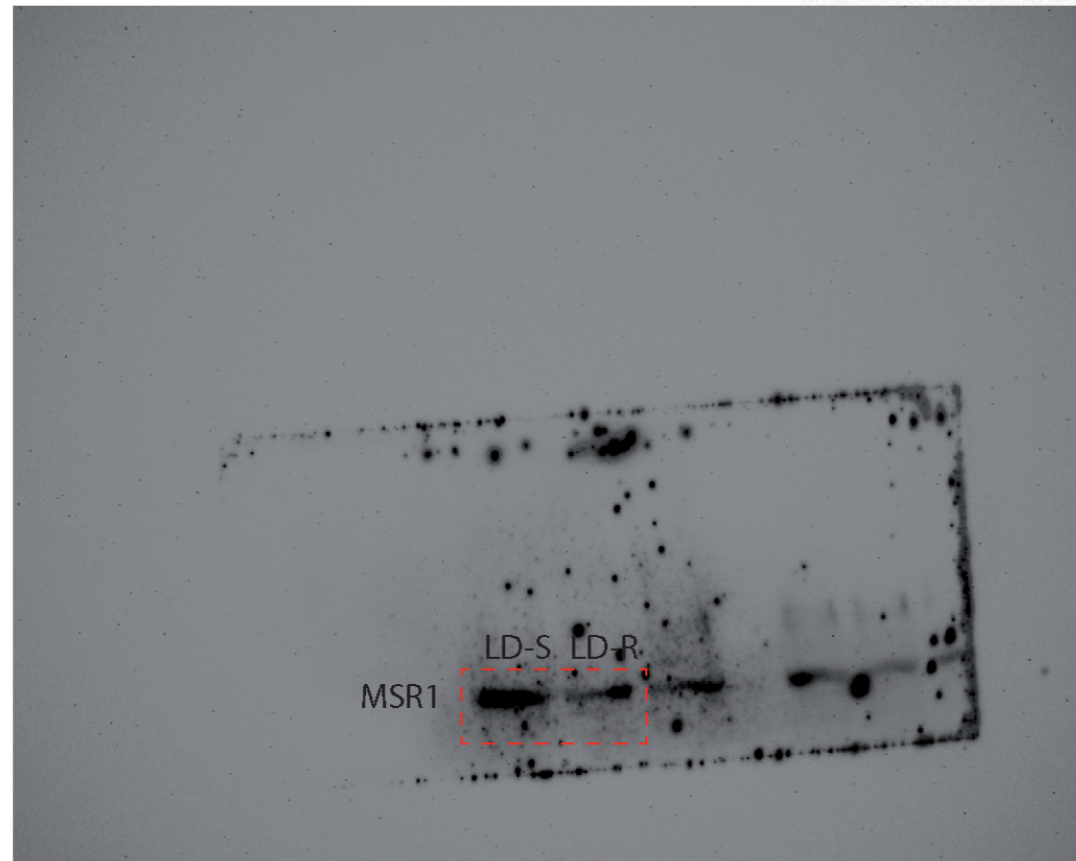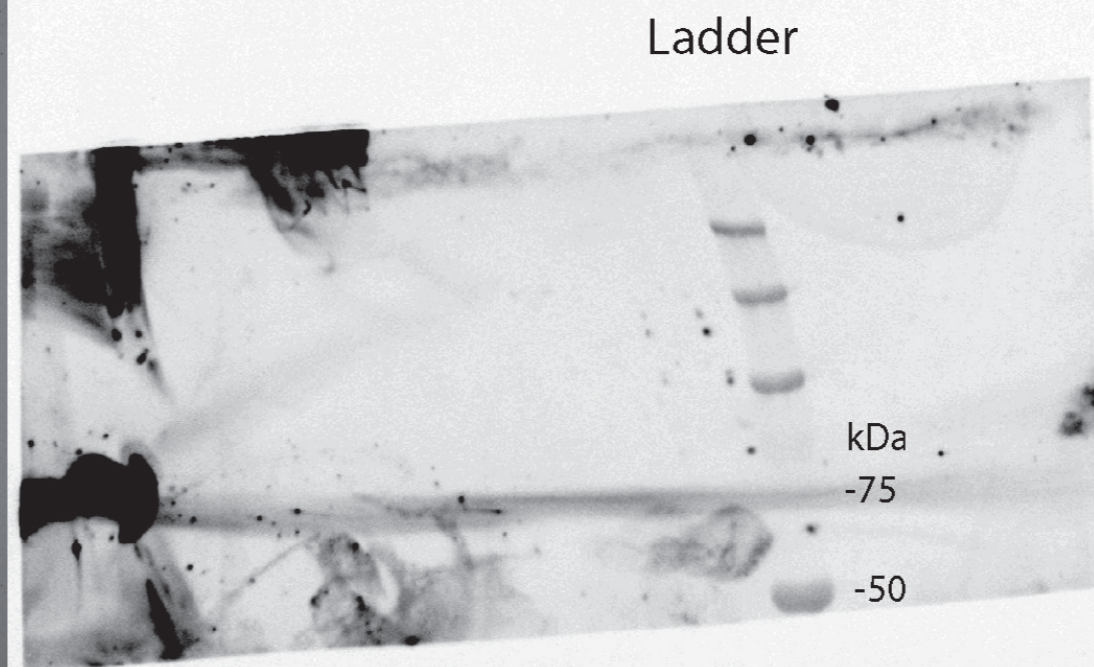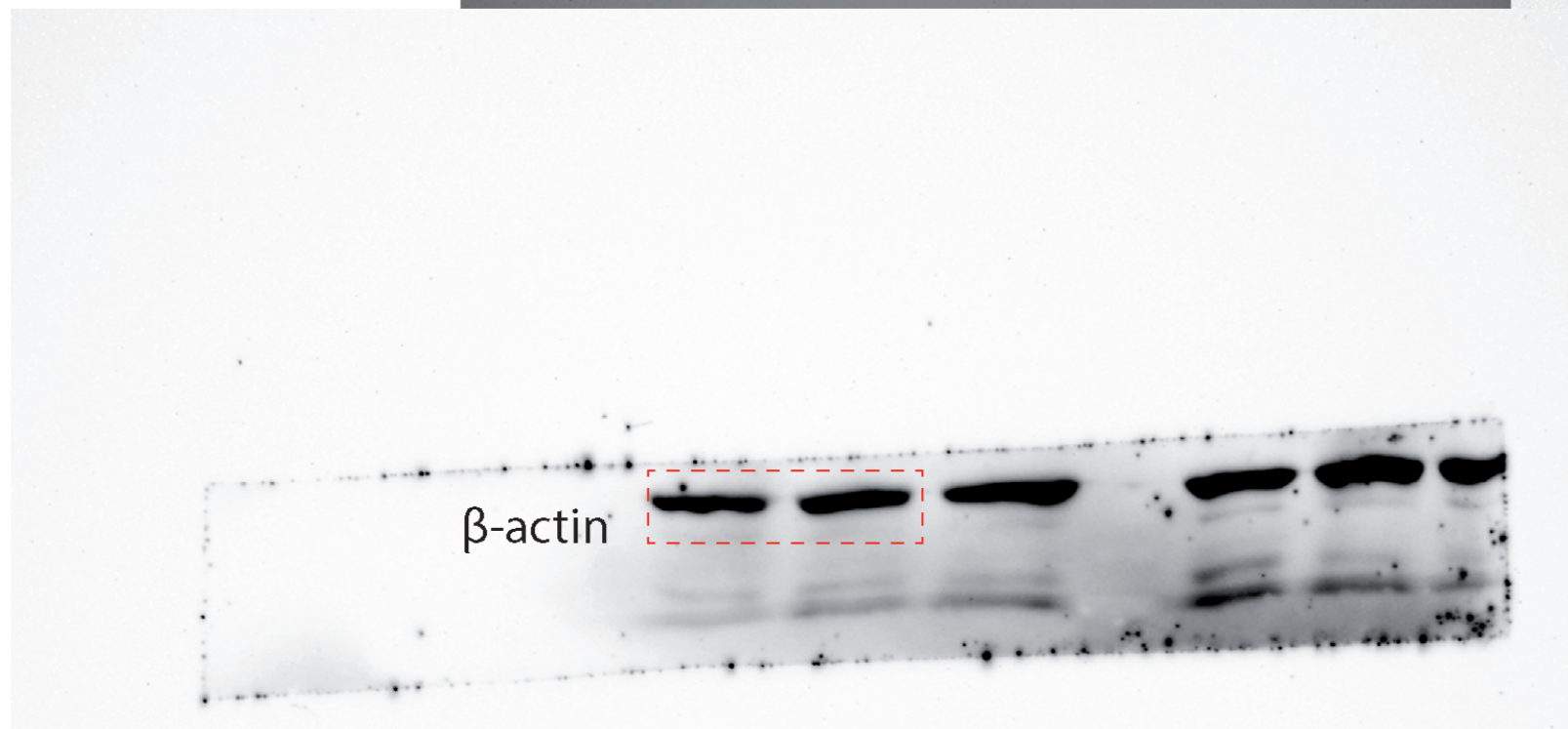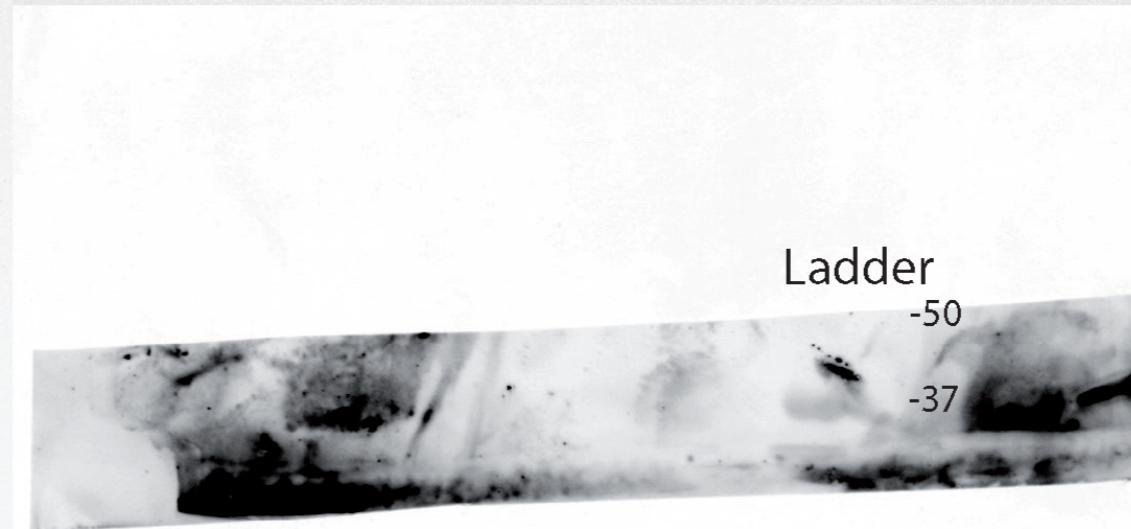

Supplement: Figure 7—source data 2. [file elife-102857-fig7-data2.zip › Figure 7 source data 1 marked/Figure 7 source data 1 marked.pdf]

E

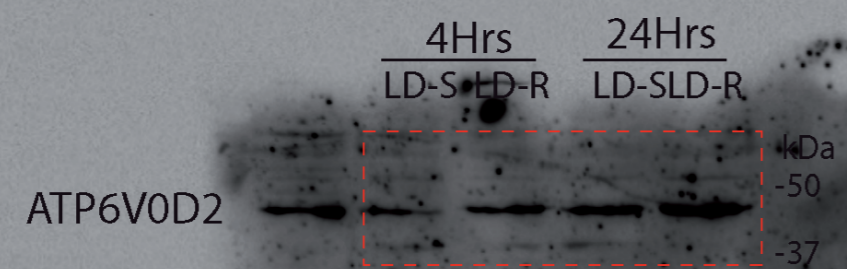

$\beta$ -actin

50  
37

Supplement: Figure 7—source data 2. [file elife-102857-fig7-data2.zip › Figure 7 source data 1 marked/Figure 7 source data 2 marked.pdf]
